# Ability of epidemiological studies to monitor HPV post-vaccination dynamics: a simulation study

**DOI:** 10.1017/S0950268823000122

**Published:** 2023-02-02

**Authors:** Mélanie Bonneault, Elisabeth Delarocque-Astagneau, Maxime Flauder, Johannes A. Bogaards, Didier Guillemot, Lulla Opatowski, Anne C. M. Thiébaut

**Affiliations:** 1 Epidemiology and Modelling of Antibiotic Evasion Unit, Institut Pasteur, 75475 Paris, France; 2 Université Paris-Saclay, UVSQ, Inserm, CESP, Anti-Infective Evasion and Pharmacoepidemiology Team, 78180 Montigny-Le-Bretonneux, France; 3 Université Paris-Saclay, UVSQ, Inserm, CESP, High Dimensional Biostatistics Team, 94807 Villejuif, France; 4 Department Epidemiology & Data Science, Amsterdam University Medical Centers, Amsterdam, Netherlands; 5 Department of Public Health, AP-HP, Paris Saclay, Medical Information, Clinical Research, 94276 Le Kremlin–Bicêtre, France

**Keywords:** Agent-based modelling, genotype interactions, human papillomavirus, observational study simulations, vaccination

## Abstract

Genital human papillomavirus (HPV) infections are caused by a broad diversity of genotypes. As available vaccines target a subgroup of these genotypes, monitoring transmission dynamics of nonvaccine genotypes is essential. After reviewing the epidemiological literature on study designs aiming to monitor those dynamics, we evaluated their abilities to detect HPV-prevalence changes following vaccine introduction. We developed an agent-based model to simulate HPV transmission in a heterosexual population under various scenarios of vaccine coverage and genotypic interaction, and reproduced two study designs: post-*vs.*-prevaccine and vaccinated-*vs.*-unvaccinated comparisons. We calculated the total sample size required to detect statistically significant prevalence differences at the 5% significance level and 80% power. Although a decrease in vaccine-genotype prevalence was detectable as early as 1 year after vaccine introduction, simulations indicated that the indirect impact on nonvaccine-genotype prevalence (a decrease under synergistic interaction or an increase under competitive interaction) would only be measurable after >10 years whatever the vaccine coverage. Sample sizes required for nonvaccine genotypes were >5 times greater than for vaccine genotypes and tended to be smaller in the post-*vs.*-prevaccine than in the vaccinated-*vs.*-unvaccinated design. These results highlight that previously published epidemiological studies were not powerful enough to efficiently detect changes in nonvaccine-genotype prevalence.

## Introduction

The discovery of human-papillomavirus (HPV) as a necessary cause in cervical cancer development has led to vaccine development to prevent cancers associated with HPV infection [1]. To date three vaccines (bivalent, quadrivalent and, more recently, nonavalent) have been introduced in populations worldwide [2]. All vaccines target at least HPV-16 and -18, which have been shown to account for about 70% of cervical cancers [3]. Following the introduction of HPV vaccines, lower prevalences of HPV-infections with the targeted genotypes (V) were demonstrated in several countries [4, 5]. However, quantifying the global impact of vaccines on the overall HPV prevalence and cervical cancer incidence is difficult. First, the measured impact is still mostly based on the prevalence of HPV infections with V-genotypes, much less with genotypes not included in the vaccine (non-vaccine, NV), and hardly at all on cancer incidence given the time it takes for the cancer to develop [6]. Second, several aspects of HPV-infection natural history such as clearance, natural immunity after infection and possible ecological interactions between genotypes remain poorly understood, which can lead to misinterpretation of observed prevalence dynamics [7, 8]. Notably, the mechanisms underlying the ecological coexistence of widely diverse HPV genotypes have not yet been elucidated [9, 10]. Some evidence indicates that coinfection by at least two genotypes (accounting for an estimated 20% to 70% of all infections) could affect viral load, cell-infection ability and/or time to clearance [11–14]. If so, the V-prevalence decline could then facilitate the spread of NV-genotypes in the population – in the case of ecological competition with V-genotypes – and subsequently increase NV-prevalence after vaccine introduction.

Detecting and anticipating vaccination impact in such a complex interacting system is challenging. To what extent do classical epidemiological studies capture these changes of dynamics? Mathematical modelling and computer simulations, which enable in silico integration of biological and epidemiological hypotheses, can help analyse the expected dynamics under hypothetical scenarios.

Herein, we aimed to identify the conditions necessary for epidemiological studies to detect HPV-prevalence changes after HPV–vaccine introduction. After reviewing observational study designs, we conducted simulations to assess HPV-postvaccine trends under the most widely used study designs.

## Methods

### Identification of epidemiological study designs

We conducted a systematic literature review to identify the epidemiological study designs used to assess NV-genotype prevalence trends after vaccination. We searched Medline databases up to 7 September 2021, with the following combination of MeSH terms: ‘humans’ AND (‘immunization programs’ OR ‘papillomavirus vaccines’) AND ‘papillomavirus infections’ AND ‘prevalence’. We classified retained epidemiological studies into two distinct designs: those comparing HPV prevalence in populations before and after vaccine introduction (hereafter called post-*vs.*-pre), and those comparing vaccinated-*vs.*-unvaccinated populations in the postvaccine era.

### Agent-based simulations

**Fig. 1.**
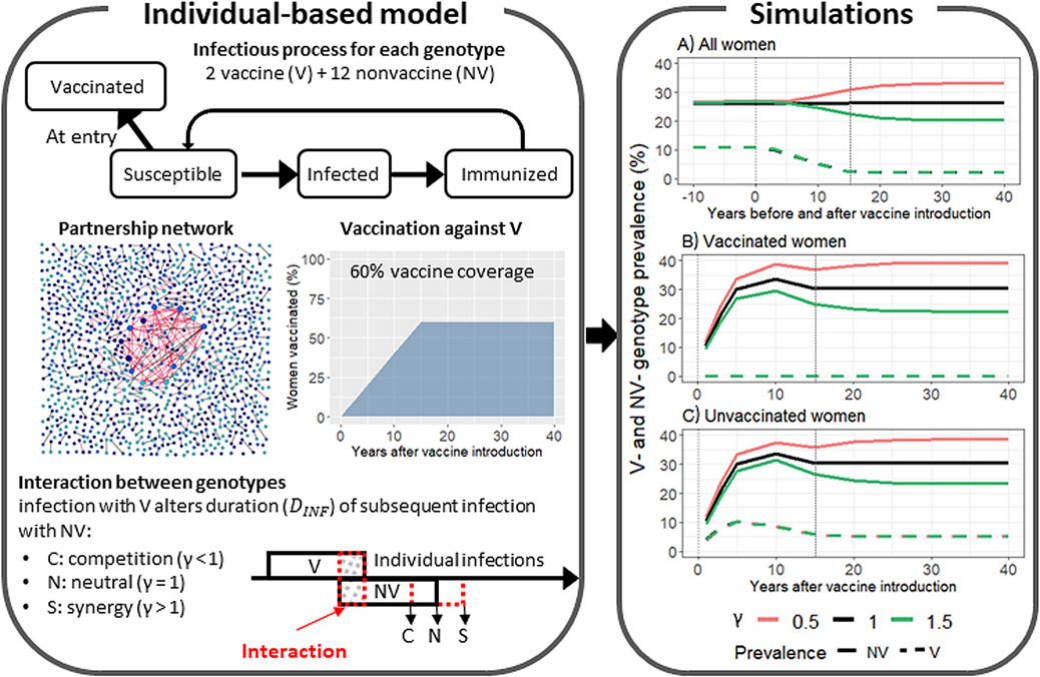
Schematic view of components of the agent-based model and simulation results of vaccine (V)- and nonvaccine (NV)-genotype prevalences over time (median values over 100 iterations) assuming 60% vaccine coverage and selected V–NV-genotype interaction strengths (*γ*): (A) all women (all ages); (B) vaccinated and (C) unvaccinated women of ages targeted by vaccination. Results from A were used for post-*vs.*-prevaccine comparisons, while results from B and C combined were used for vaccinated-*vs.*-unvaccinated comparisons. The dashed vertical line at 15 years indicates when all age cohorts have been offered the vaccine. For V genotypes, the 3 curves according to interaction strength overlapped.

We simulated the transmission dynamics of V- and NV-genotype prevalences pre- and postvaccination using a stochastic agent-based model encompassing transmission of multiple HPV genotypes over a heterogeneous sexual network. The model description is schematised in Figure 1 and detailed elsewhere [15]. Briefly, we simulated a heterosexual population of 800,000 individuals, each characterised by his/her age, sex, sexual activity class (3 categories: 1, 2–3 or >3 partners per year) determining the parameters ruling partner acquisition, partnership duration (concurrent partnership is not allowed) and time without partnership, and infection status for each genotype (4 categories: susceptible, infected, naturally immune or vaccinated against a V genotype). Individuals of both sexes enter the population at age 15 years, leave it at age 30, and evolve every week in between. We modelled individually HPV transmission of 14 high-risk genotypes: 2 for V types, and 12 for NV types. When a contact occurred between two individuals, the model allowed either single transmission of one genotype, or simultaneous transmission of multiple genotypes in case of a multi-infected partner. Acquired immunity was also genotype-specific. After acquisition and clearance of a given genotype, an individual was assumed to be totally protected over a duration of 12 weeks on average to that specific genotype, not affecting acquisition of others. We assumed genotype-specific natural immunity during a defined time and introduced vaccination of <15-year-old women before their first partner, assuming vaccine provides full immunity against infection with V genotypes. The partnership process was calibrated against behavioural data from a French nationwide survey [16], and the HPV-transmission process was calibrated against the prevaccine prevalence observed in a US study [17].

### Interactions between genotypes

We assumed that an individual already infected with a V genotype has the duration of a subsequent NV-genotype infection modified by a multiplicative factor *γ*, denoting the strength of interaction (Fig. 1). In addition to the neutral interaction scenario (*γ* = 1), we considered two competitive scenarios, either stronger (*γ* = 0.5) or weaker (*γ* = 0.9), in which V genotypes partially limit infection with NV genotypes, and two synergistic scenarios, either stronger (*γ* = 1.5) or weaker (*γ* = 1.1), in which V genotypes facilitate infection with NV genotypes. For each scenario, we calibrated the transmission-probability parameter of NV genotypes to reproduce reported NV-infection prevalences before vaccine introduction (Supplementary paragraph S1) [17].

### Computer simulations

The model was developed in C++, statistical analyses and graphics were computed using R (version 3.5.2). Simulations were run on the TARS cluster of the IT Department, Institut Pasteur, Paris.

### Statistical analyses

Because vaccine introduction was carried out by age cohort, the statistical analyses were restricted to the age groups offered the vaccine (i.e. women aged 15, 15–17, 15–19, 15–24 or all aged 15–29 years respectively 1, 3, 5, 10 or ≥15 years after vaccine introduction, as shown Supplementary Fig. S1, paragraph S2.1). Similarly, the age-matched prevaccination population was selected among those targeted by vaccination. From NV and V prevalences, we deduced the prevalence difference between the two compared populations according to post-*vs.*-prevaccine or vaccinated-*vs.*-unvaccinated study design. We also calculated the total sample size (summing post and prevaccine samples or vaccinated and unvaccinated samples respectively) required to detect statistically significant differences between prevalences at the 5% significance level and 80% power (see Supplementary paragraph S2.2 for detailed formulas). We also calculated the minimal size of a prevaccine sample needed retrospectively to ensure sufficiently powered comparison with a postvaccine sample to be collected prospectively (Supplementary paragraph S2.3). Median values and 90% empirical intervals over 100 independent simulations for each scenario are reported. These analyses were repeated by sexual activity group (1 to 3 partners or >3 partners in the past year) to better understand how prevalence variations were distributed in the population.

### Complementary analyses

As a sensitivity analysis, we tested fine-grained ranges of strengths of competitive (*γ* = 0.5, 0.6, 0.7, 0.8 and 0.9) and synergistic (*γ* = 1.1, 1.2, 1.3, 1.4 and 1.5) interactions (see Supplementary paragraph S1).

We also analysed a single NV genotype to help disentangle the consequences of genotypic interactions from those related to the analysis of genotype groups when stratifying on sexual activity groups (details in Supplementary paragraph S2.4).

## Results

### Literature review

**Fig. 2.**
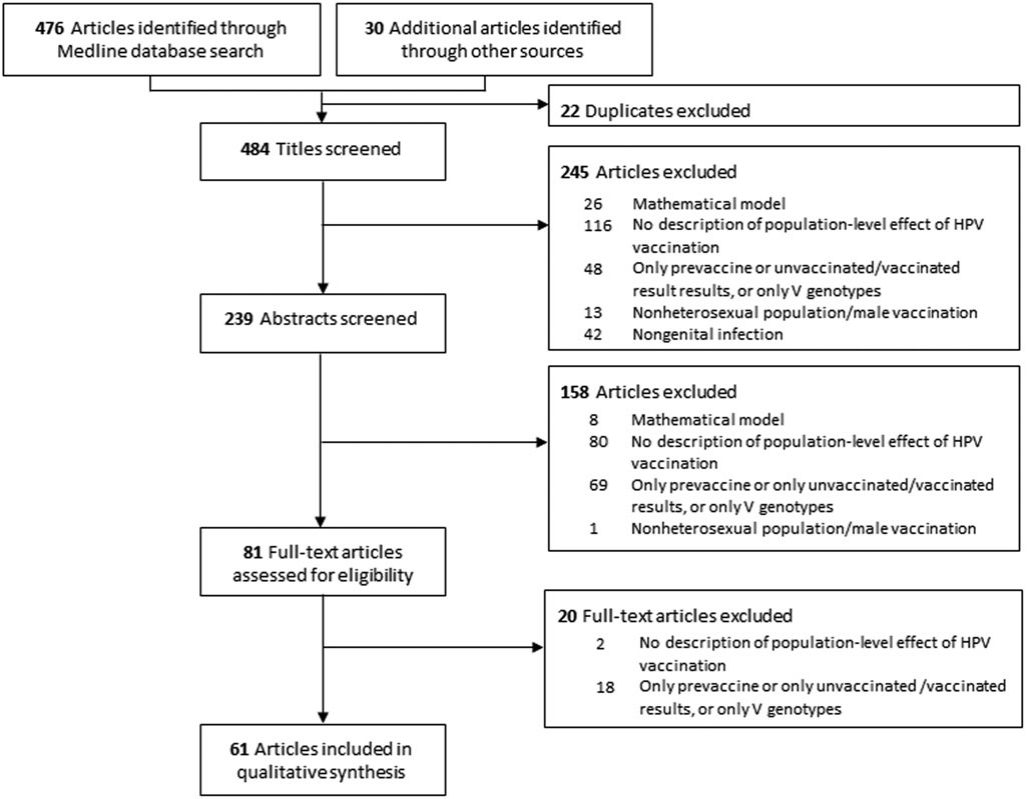
PRISMA (Preferred Reporting Items for Systematic Reviews and Meta-Analyses) flow diagram.

**Fig. 3.**
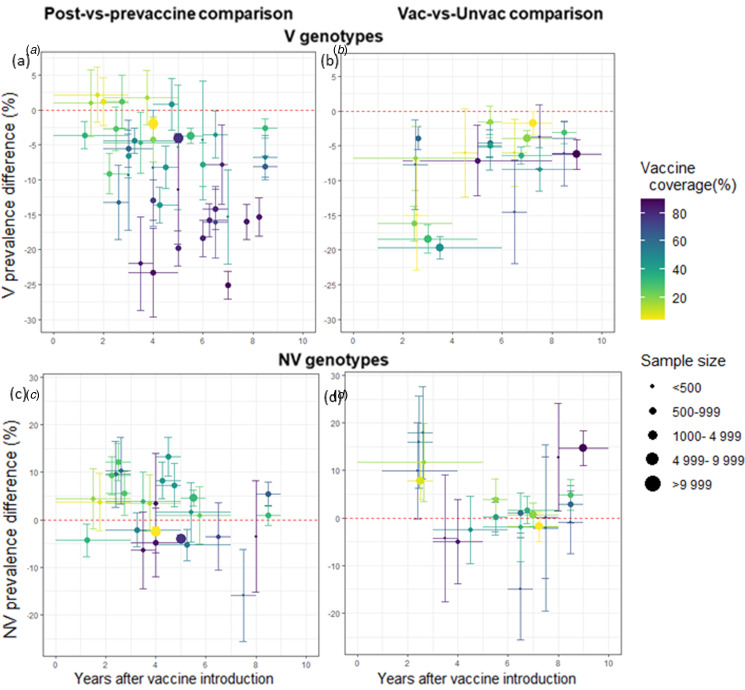
Prevalence differences reported in observational studies for V (a and b) and NV (c and d) high-risk genotypes by study design: post-*vs.*-prevaccine (a and c) and vaccinated-*vs.*-unvaccinated (b and d), according to vaccine coverage (colour gradient from yellow, low coverage to purple, high coverage) and sample size (dot size from small, low sample size to large, large sample size). Vertical intervals correspond to reported 95% confidence intervals while horizontal lines correspond to the time span covered.

We identified 506 potentially eligible articles and included 61 in our review (Fig. 2). Of these, we extracted 20 post-*vs.*-pre comparisons and 32 vaccinated-*vs.*-unvaccinated comparisons, as described in Supplementary Tables S3 and S4, respectively (paragraph S3.1). Their results are synthesised in Figure 3. For V genotypes (Figs 3a and b), most studies of both designs showed a significant prevalence decrease following vaccine introduction. Moreover, for post-*vs.*-pre studies (Fig. 3a), V-prevalence differences tended to be greater in magnitude with higher vaccine coverage and with longer time since vaccine introduction. This trend was less clear for the vaccinated-*vs.*-unvaccinated comparison (Fig. 3b) or for NV genotypes in both comparisons (Figs 3c and d).

### Monitoring of V-genotype prevalence trends

**Fig. 4.**
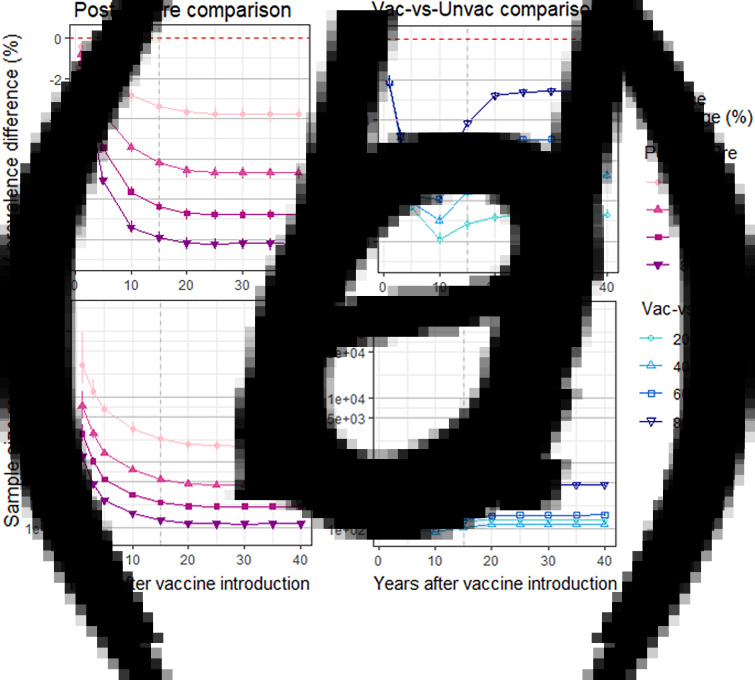
Prevalence differences for V genotypes (a and b) and corresponding sample sizes (c and d) over time according to epidemiological study design (a and c: post-*vs.*-prevaccine (post-*vs.*-pre), b and d: vaccinated *vs*. unvaccinated (vac-*vs.*-unvac) comparisons) and vaccine coverage under the neutral interaction scenario (*γ* = 1). The dashed vertical line at 15 years indicates when all age cohorts have been offered the vaccine. Results shown are medians and 90% empirical intervals over 100 simulations.

Starting with V genotypes, simulation results were identical regardless of interaction strength (Fig. 1, right panel). However, the choice of epidemiological study design resulted in distinct V-prevalence–trend differences over time (Figs 4a and b).

For post-*vs.*-pre comparisons, although the magnitude of prevalence differences became greater, the successive curves obtained were similarly shaped as immunisation coverage rose (Fig. 4a). All prevalence differences stabilised after 25 years, reaching −10.7% in median for 80% vaccine coverage. Total sample sizes were then <500 women for any vaccine coverage ≥40% (Fig. 4c).

In contrast, for vaccinated-*vs.*-unvaccinated comparisons, prevalence differences of similar magnitudes were detectable as early as 5 years postvaccine introduction (medians ranging between −7.53% and −8.32%) with median total sample sizes between 99 and 159 women within the age groups offered the vaccine (Figs 4b and d). Prevalence differences continued to increase until 10 years for vaccine coverage ≤60%. Thereafter, absolute differences decreased sharply, until levelling off after 25 years: the higher the vaccine coverage, the smaller the median magnitude of prevalence difference (respectively, −2.62% and −8.72%, for 80% and 20% vaccine coverage) and, consequently, the larger the sample size required (465 and 137 women, respectively).

### Monitoring of NV-genotype prevalence trends

**Fig. 5.**
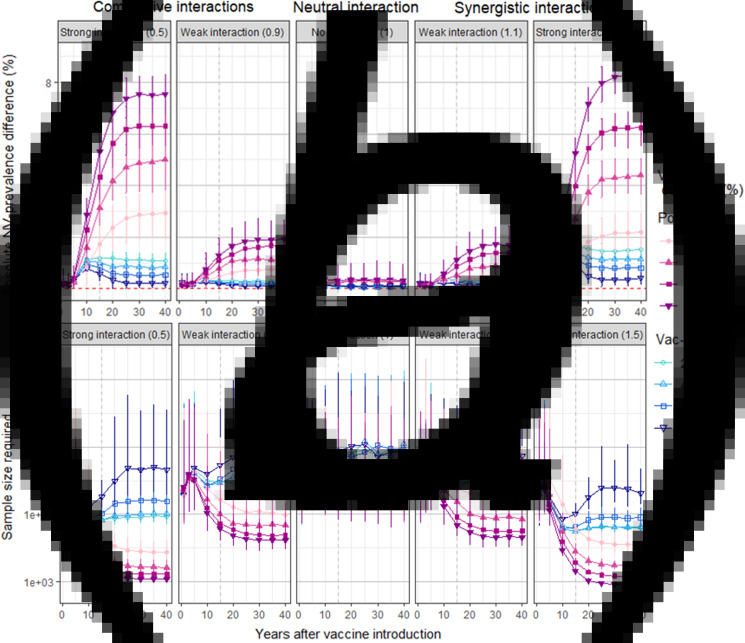
Absolute prevalence-difference values for NV genotypes (a) and corresponding sample sizes (b) over time according to strength of competitive, neutral and synergistic interactions (*γ*), epidemiological study design (post-*vs.*-pre or vaccinated-*vs.*-unvaccinated) and vaccine coverage. Dashed vertical lines at 15 years indicate when all age cohorts have been offered the vaccine. Median values and 90% empirical intervals over 100 simulations are shown. For synergistic values, prevalence differences are negative; they are presented here as absolute values for ease of comparability.

Unlike V genotypes, NV-genotype–prevalence trends over time strongly depended on the interaction strength (Fig. 1, right panel, and Supplementary Fig. S2 in paragraph S3.2): after vaccine introduction, NV prevalences deviated from the neutral scenario, increasing for competitive interaction (*γ* < 1) and decreasing for synergistic interaction (*γ* > 1). As shown in Figure 5a, these trends were more marked for post-*vs.*-pre than vaccinated-*vs.*-unvaccinated study design, and those with stronger than weaker interaction strength, respectively in competition (i.e. *γ* = 0.5 *vs.* 0.9) and synergy (i.e. *γ* = 1.5 *vs.* 1.1). For post-*vs.*-pre comparisons, similarly shaped curves were obtained over time, plateauing after 30 years, regardless of interaction strength and vaccine coverage (Fig. 5a). Moreover, at a given interaction strength, the higher the immunisation coverage, the greater the median magnitude of prevalence difference. For vaccinated-*vs.*-unvaccinated comparisons, absolute prevalence differences increased until 10 years postvaccine introduction, when the age cohorts that have been offered the vaccine included the age category 20–24 years, coinciding with peak HPV-prevalence, and decreased thereafter. Opposite to the post-*vs.*-pre design, prevalence differences of greater magnitude were obtained for lower immunisation coverage. Critically, in both study designs and interaction scenarios, prevalence differences were hardly visible within the first 5 years postvaccine introduction.

Because the prevalence differences were more pronounced for post-*vs.*-pre than vaccinated-*vs.*-unvaccinated comparisons, required sample sizes were smaller for the former than the latter (Fig. 5b and Supplementary Figs S2D–F). For example, 15 years postvaccine introduction, under stronger interaction scenarios and 60% vaccine coverage, median prevalence differences for competitive and synergistic interactions were, respectively, 5.64% and −5.45% for the post-*vs.*-pre comparison *vs.* 0.59% and −0.90% for the vaccinated-*vs.*-unvaccinated comparison, starting from a prevalence of 26.5% before vaccine introduction. The corresponding median total sample sizes for competitive and synergistic interactions were, respectively, 2039 and 1906 for post-*vs.*-pre comparison and 208,373 and 67,578 women for vaccinated-*vs.*-unvaccinated comparison. For the post-*vs.*-pre comparison, allowing unbalanced sampling between the two groups slightly reduced the median sample sizes in the prevaccine era for competitive and synergistic interactions, respectively, 860 and 915 women (as compared to 2039/2 and 1906/2, see details in Supplementary paragraph S3.3).

### Targeting specific sexual activity groups

**Fig. 6.**
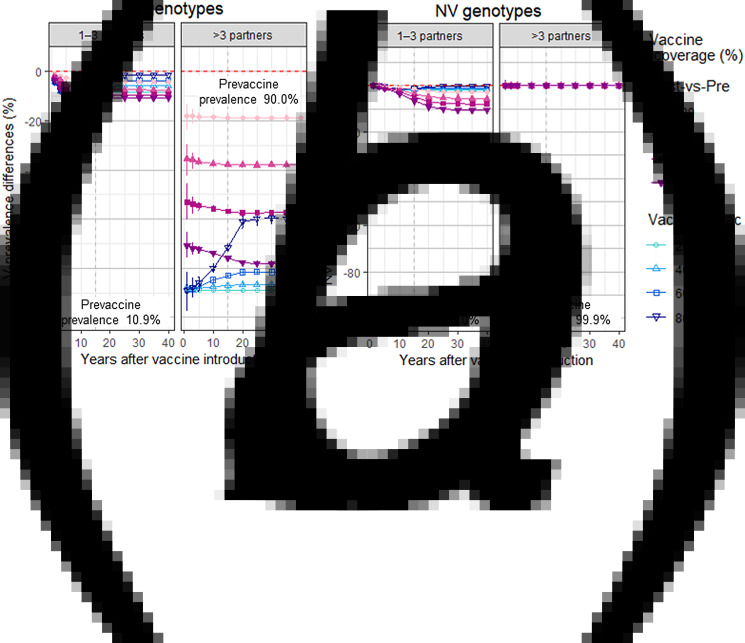
Prevalence differences for (a) V- and (b) NV-genotypes over time under strong synergistic interaction (*γ* = 1.5), according to the individual's number of partners during the past year, epidemiological study design (post-*vs.*-pre or vaccinated-*vs.*-unvaccinated) and vaccine coverage. Dashed vertical lines at 15 years indicate when all age cohorts have been offered the vaccine. Median values and 90% empirical intervals over 100 simulations are reported; V- or NV-genotype prevalence before vaccine introduction in each sexual activity group is specified on each graph.

Finally, stratifying on individual sexual activity, we found that the magnitude of V-genotype prevalence differences were at least five times greater for more connected women (>3 partners in the past year) than for less connected women (Fig. 6a). In contrast, prevalence differences for all NV genotypes taken together were detectable only for less connected women, under synergistic (Fig. 6b) as well as competitive interaction (results not shown). This observation did not hold true when focusing on a single NV genotype: prevalence differences were then more pronounced among individuals with >3 partners than among individuals with 1–3 partners, regardless of the epidemiological study design and vaccine coverage (Fig. S4 and details in Supplementary paragraph S3.4).

## Discussion

Our epidemiological literature review identified two study designs used to assess HPV-prevalence changes following vaccination: prevalence comparisons of the post-*vs.*-prevaccine eras and vaccinated-*vs.*-unvaccinated women in the postvaccine era. From the simulation of a realistic agent-based model simulating V- and NV-genotype transmissions among 15–30-year olds over a heterogeneous human network, we observed that both designs enabled early detection of V-genotype–prevalence reduction under most scenarios of genotypic interaction and vaccine coverage. However, longer times after vaccine introduction and larger sample sizes were required to detect changes in NV-genotype prevalence than for V genotypes, and post-*vs.*-pre comparisons generally performed better than vaccinated-*vs.*-unvaccinated comparisons.

In accordance with phase-3 randomised controlled trials of HPV vaccines [18, 19], almost all observational studies carried out so far have shown prevalence reductions for genotypes targeted by HPV vaccination (Fig. 3) [4, 20], with differences ranging between 1.6% and 27.0% for post-*vs.*-pre designs (Supplementary Table S3), and between 0.1% and 19.6% for vaccinated-*vs.*-unvaccinated designs (Supplementary Table S4). Before comparing our simulation results with those of observational studies, we should acknowledge important discrepancies between these studies, including HPV prevalence before vaccination, sample characteristics, method of genotype detection, number of genotypes considered, etc. For post-*vs.*-pre designs, our simulation results are consistent with the trends observed in our literature review (Fig. 3a). Our results are particularly consistent with those of Purriños-Hermida *et al*. [21] who reported 3.5% and 6.1% prevalence reductions, respectively 6 and 9 years after vaccine introduction, corresponding to 43% and 53% vaccine coverage. In comparison, we found medians of 3.7% and 5.4%, respectively 5 and 10 years after vaccine introduction under 40% vaccine coverage, and 7.6% 10 years after under 60% vaccine coverage. Our results for the vaccinated-*vs.*-unvaccinated design are also comparable: they found an 8.4% prevalence difference about 8 years after vaccine introduction, with 47% vaccine coverage [21], while we obtained 8.9% after 10 years under 40% vaccine coverage.

For NV genotypes, epidemiological studies have yielded contradictory results for the direction and magnitude of prevalence differences (Figs 3c and d). Higher NV prevalences were sometimes reported in the comparison group (postvaccine or vaccinated) than in the reference group (prevaccine or unvaccinated, respectively) for all high-risk NV genotypes combined [4, 22, 23], all except HPV-31, -33, -45 [24], all except HPV-45 [25] or some specific NV genotypes, e.g. HPV-39, -51, -52, -56 and -59 [26]. However, smaller NV prevalences in the comparison *vs.* reference group were also reported, especially for HPV-31, -33 and/or -45 [4, 21, 22, 24, 27–30] or for all high-risk NV genotypes [28, 29, 31]. Compared to our model simulations, most studies had smaller sample sizes than the required number of subjects we estimated and they were conducted only a few years after vaccine introduction. Even the most recent studies, despite longer times since vaccine introduction (up to 8–10 years), had sample sizes too small to detect significant NV-prevalence variation, if it exists [21, 32, 33]. It seems important to note here that several studies reported a rapid reduction in the prevalences of HPV-31, -33 and -45 after vaccine introduction [21, 22, 24, 29]. Nevertheless, this rapid reduction which differs from that observed in our simulations is most likely a direct protective effect of the vaccine via cross-reactivity, i.e. vaccine-induced antibodies able to neutralise these NV genotypes [18, 27, 28, 34] but phylogenetically related to V genotypes [35]. Here, to keep the model as simple as possible and focus on between genotypes interactions, this mechanism was not considered. Taking cross-protection into account would have possibly led to different post-vaccination trends, with a rapid decrease in NV prevalence, the magnitude depending on the assumed strength of the vaccine's cross-protection against these types. In such case, the longer-term effect of interactions, observed here 10 years after vaccine introduction, should be observed equally but with a lower magnitude, due to previous decrease of NV prevalence associated with vaccine cross-protection.

The estimated time to detect NV-prevalence variations can seem unrealistically long in comparison with other pathogens, notably *Streptococcus pneumoniae* for which serotype replacement occurred quickly after vaccine introduction [36]. Several differences should be highlighted here. First, HPV being transmitted through sexual contacts, incident cases are much less frequent than for common bacterium like pneumococcus. Consequently, ecological variations are not expected to occur on the same magnitude and time scale. Second, because *S. pneumoniae* carriage may start as early as birth, vaccinated individuals can be immediately exposed to infection. In contrast, because HPV vaccination is recommended at a young age, before the first risk of infection, the vaccination–infection interval can be much longer. This time could be shortened if vaccination were to be achieved simultaneously for the whole population. Nevertheless, our simulation results are in accordance with recent results, based on the modelling of two genotypes interacting through infection-induced cross-immunity, showing expected NV-genotype–prevalence variations >10 years postvaccine introduction [37].

To compensate for the limited time elapsed since vaccine introduction, some observational studies attempted to gain statistical power by targeting populations at higher risk of exposure to HPV [30, 38, 39]. In particular, studies conducted in sexual health clinics may include more women with higher-risk sexual behaviours (e.g. a higher number of partners). In our model simulations, V-genotype–prevalence differences were more pronounced in highly active (>3 partners) than less connected women. That finding is in line with *post hoc* analyses from a community randomised trial in Finland that compared HPV-vaccinated *vs.* nonvaccinated women, using *Chlamydia trachomatis*-infection status as a surrogate for sexual activity [39]. Those authors reported that V-genotype–prevalence ratios per genotype were <1 and more marked for women positive for *C. trachomatis* than negative. For a few specific NV genotypes, they observed prevalence ratios >1, especially for *C. trachomatis*-positive subjects. Similarly, we observed greater prevalence differences in more *vs.* less connected individuals when we considered a single rather than all NV genotypes. Grouping NV genotypes resulted in such high prevaccine prevalence in more connected individuals that vaccine-induced decrease in V-genotype prevalence hardly changed it.

Our results should be interpreted in light of the limitations of the agent-based model we used, as previously underlined [15]. First, the lack of knowledge regarding the mechanisms of immunity and interactions among HPV genotypes, mechanistic assumptions had to be made in the model, potentially affecting our results on post-vaccine prevalence and vaccine-impact. On the one hand, reducing the duration of immunity may increase the magnitude of variations in NV prevalence and consequently would reduce the required sample size. However and importantly, this assumption does not impact the dynamics of these trends neither the time required to detect a significant change. On the other hand, alternative interaction mechanisms, e.g. assuming that interaction affect acquisition risk or that they have a symmetric pattern between V and NV types, may also be worth investigating. This was done in a previous work, where we found that, for a given interaction strength, variations in prevalence were of similar magnitude whether interaction affected the acquisition or the duration of infection [15]. This analysis suggests that considering other interactions mechanisms would not affect the main results presented here. In the same previous study, compared to unidirectional interaction, differences of V-genotypes prevalences were more pronounced under symmetrical interaction but differences of NV-genotypes prevalences were of similar magnitude [15]. Another important hypothesis was the assumption of homogeneous interactions among V and NV genotypes. Considering heterogeneous interactions depending on specific types from one group or the other would have been more realistic; however, the lack of available data impedes to take accounted such phenomenon. Grouping all NV genotypes possibly masked different variations for specific genotype or NV subgroup: considering such heterogeneity would have enabled vaccine introduction to be followed by heterogeneous ecological trends depending on the specific genotypes. Second, for simplicity, we did not consider catch-up vaccination that is made available in some countries for older girls before onset of their sexual activity. Vaccinating multiple age cohorts was shown to have greater impact on HPV prevalence than vaccinating single cohorts [4]; it could indeed result in shorter time between vaccine introduction and detectable NV-prevalence variations.

To conclude, our results suggest that detecting potential increase of HPV-NV genotypes prevalence (so-called genotype replacement) requires epidemiological studies of large sizes and sufficiently long after vaccine introduction. Consequently, observational studies published so far could be underpowered to show any statistically significant NV-prevalence difference either between pre- and postvaccine eras or between vaccinated and unvaccinated women. Further knowledge on HPV-infection natural history is warranted to derive more robust results from our simulation model and anticipate trends after the recent introduction of a nine-valent vaccine.

## Data Availability

All materials (data and codes) needed to replicate the findings of the article is available at https://github.com/mbonneault/Monitor_HPV_dynamics.

## Supplementary material

For supplementary material accompanying this paper visit http://doi.org/10.1017/S0950268823000122. click here to view supplementary material
