# Supplementary material for: Ability of epidemiological studies to monitor HPV post-vaccination dynamics: a simulation study
Source: Epidemiol Infect. 2023 Feb 2;151:e31. doi: 10.1017/S0950268823000122 (PMC9990403; doi:10.1017/S0950268823000122)
Supplement: Supplementary file 1 [file S0950268823000122sup001.docx]

**Epidemiology and Infection**

Ability of epidemiological studies to monitor HPV post-vaccination dynamics: a simulation study

Mélanie Bonneault, Elisabeth Delarocque-Astagneau, Maxime Flauder, Johannes A. Bogaards, Didier Guillemot, Lulla Opatowski and Anne C.M. Thiébaut

Supplementary Material

Contents

[S1. Calibration of interaction scenarios 3](#_Toc119682000)

[S2. Complementary information on analysis of simulated results 3](#_Toc119682001)

[S2.1 The study population 3](#_Toc119682002)

[S2.2 Calculation of required sample size 4](#_Toc119682003)

[S2.3 Minimum sample size of an existing prevaccine study 5](#_Toc119682004)

[S2.4 Analyzing the dynamics according to sexual activity for a single NV genotype 5](#_Toc119682005)

[S3. Complementary results 6](#_Toc119682006)

[S3.1 Results of the systematic review 6](#_Toc119682007)

[S3.2 Sensitivity analysis of the impact of strength of interaction 19](#_Toc119682008)

[S3.3 Minimum sample size of an existing prevaccine study 20](#_Toc119682009)

[S3.4 Analyzing the dynamics according to sexual activity for a single NV genotype 21](#_Toc119682010)

[S4. References 23](#_Toc119682011)

Figures

**Figure S1.**Proportions of women in the modelled population whose age is targeted by vaccination, according to the number of years since vaccine introduction. 4

**Figure S2.** NV-genotype–prevalence differences (A–C in absolute values) and corresponding sample sizes (D–F) according to interaction strength at 5 (A & D), 10 (B & E) or 20 years (C & F) after vaccine introduction. 19

**Figure S3.** Sample size in prevaccine era for post-vs-prevaccine design according to time since vaccine introduction, strength of interaction and vaccine coverage. 20

**Figure S4.** NV-genotype–prevalence difference (A, B) and corresponding sample size (C, D) over time in the case of strong competitive (0.5; A, C) and synergistic (1.5; B, D) interactions (γ) according to the individual’s number of partners during the past year, the epidemiological study design (post-vs-pre or vaccinated-vs-unvaccinated), and vaccine coverage. 21

Tables

**Table S1.** Results of calibration of each interaction scenario on prevalences by age 3

**Table S2.** Values of proportionality parameter k according to vaccine coverage 5

**Table S3.** Characteristics of the studies included in the systematic review (original articles only): comparison of HPV-genotype prevalences among subjects in the pre- and postvaccine eras 7

**Table S4.** Characteristics of the studies included in the systematic review (original articles only): comparison of HPV-genotype prevalences among vaccinated and unvaccinated subjects in the postvaccine era 13

# Calibration of interaction scenarios

For each interaction-strength scenario considered in the main analysis (γ = 0.5, 0.9, 1, 1.1, 1.5) and the sensitivity analysis (γ = 0.6, 0.7, 0.8, 1.2, 1.3, 1.4), the probability of NV-genotype transmission was independently calibrated against reported prevalences of HPV-NV genotypes by age category.[1] We chose the parameter minimizing the sum of squared differences between simulated and real prevalence data. Calibration results are reported in Table S1.

**Table S1.** Results of calibration of each interaction scenario on prevalences by age

| **Interaction strength ()** | **Estimated transmission probability parameter of NV genotypes** | **Minimized sum**  **of squares** |
| --- | --- | --- |
| 0.5 | 0.570 | 0.0027 |
| 0.6 | 0.355 | 0.0029 |
| 0.7 | 0.260 | 0.0031 |
| 0.8 | 0.210 | 0.0037 |
| 0.9 | 0.160 | 0.0037 |
| 1.0 | 0.125 | 0.0038 |
| 1.1 | 0.112 | 0.0044 |
| 1.2 | 0.098 | 0.0048 |
| 1.3 | 0.088 | 0.0052 |
| 1.4 | 0.075 | 0.0058 |
| 1.5 | 0.072 | 0.0062 |

# Complementary information on analysis of simulated results

## The study population

Because vaccine introduction is carried out by age cohort, the analysis at any given time in the simulations was restricted to the age categories offered the vaccine in the comparison and reference groups at that time. As a consequence, proportions of the population involved in the analysis increase with time since vaccine introduction, as shown in Figure S1.

| 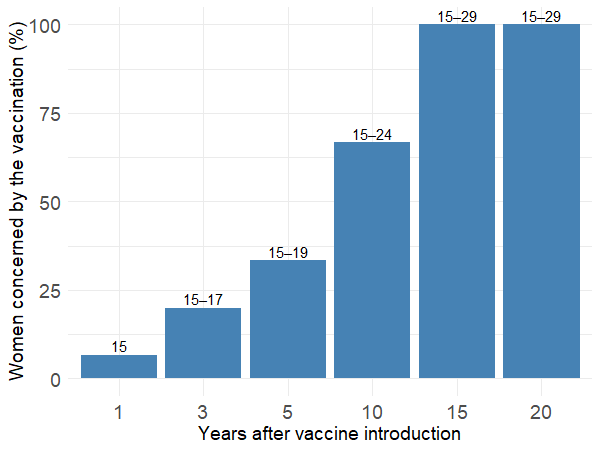 |
| --- |
| **Figure S1.**Proportions of women in the modelled population whose age is targeted by vaccination, according to the number of years since vaccine introduction. Because vaccination is introduced by age cohort, the proportion of women corresponds to that of women of the relevant ages specified above the bars (in years). |

## Calculation of required sample size

Our sample-size calculation was based on a two-sided test for the difference of proportions between two independent populations.[2] We report the total sample size $n_{0}$ + $n_{1},$ where $n_{0}$ is the number of women in the reference group (prevaccine or unvaccinated in our case) and $n_{1}$ is the number of women in the comparison group (postvaccine or vaccinated, respectively). Allowing for unequal sample sizes, those numbers were obtained as follows:

$n_{0}=k\times n_{1}$and $n_{1}=\frac{k+1}{k}\frac{(Z_{\alpha/2} -Z_{1-\beta})^{2}}{4(arcsin\sqrt{P_{1}}-arcsin\sqrt{P_{0}})^{2}}$

where *k* is a proportionality parameter reflecting the difference in size between the two groups, $Z_{\alpha/2}$ and $Z_{1-\beta}$ are quantiles of the normal distribution (here we chose significance level α = 0.05 and statistical power $1-\beta$ = 80%), *P*_0_ is the prevalence in the reference group and *P*_1_ the prevalence in the comparison group.

In the post-vs-prevaccine comparison, we first considered equal sample sizes $n_{0}$ and $n_{1}$, so$k=1$. In the vaccinated-vs-unvaccinated comparison, we adapted the value of *k* according to vaccination coverage (Table S2).

**Table S2.** Values of proportionality parameter k according to vaccine coverage

| Proportionality | Vaccine coverage (%) | | | | | | | |
| --- | --- | --- | --- | --- | --- | --- | --- | --- |
| parameter | 20 | 30 | 40 | 50 | 60 | 70 | 80 | 90 |
| *k* | 4 | 2.3 | 1.5 | 1 | 0.67 | 0.43 | 0.25 | 0.11 |

## Minimum sample size of an existing prevaccine study

Like the vaccinated-vs-unvaccinated comparison, the post-vs-prevaccine comparison could rely on samples of unequal size. Indeed, for the post-vs-prevaccine comparison, one must *a posteriori* find an existing sample that was collected in the prevaccine era, regardless of the size of the postvaccine population to be recruited. Our question was then to determine the minimal required size of this prevaccine sample, $n_{0,min}$. We proceeded as follows:

$n_{0}=k\times n_{1}=(k+1$*)* $\theta^{2}$ with $\theta^{2}=\frac{(Z_{\alpha/2} -Z_{1-\beta})^{2}}{4(arcsin\sqrt{P_{1}}- arcsin\sqrt{P_{0}})^{2}}$

As $k>0$ ⇒$n_{0,min}= \theta^{2} \mathrm{and} k=\frac{n_{0,min}}{\theta^{2}}-1$

## Analyzing the dynamics according to sexual activity for a single NV genotype

In order to disentangle mechanisms at stake and complement the analysis by sexual activity, we ran simulations while focusing on a single genotype (instead of considering the sum of all 12 NV genotypes). It is important to note here that the individual genotype prevalence was not calibrated, since calibration was performed on the total prevalence of all NV genotypes. We assumed that the NV genotype of interest had the same transmission parameter, infection duration and interaction strength as the one estimated for the whole group. Hence, the results obtained should be interpreted in the light of that limitation and only be considered as an average behavior for any NV genotype.

# Complementary results

## Results of the systematic review

61 articles met our inclusion/criteria for this systematic review. Precisely, inclusion criteria were studies describing the effect of HPV vaccines on the prevalence of NV genotypes at the population level. Exclusion criteria were:

- Articles presenting a mathematical modeling study only (and no data)
- Papers without description of population-level effect of HPV vaccination
- Articles not allowing comparisons, i.e., presenting data on prevaccine only without postvaccine prevalence, or in unvaccinated individuals only but not in vaccinated individuals, or conversely in vaccinated individuals only but not in unvaccinated individuals
- Articles presenting results on V genotypes only but none on NV genotypes
- Articles focused on HPV prevalence in non-heterosexual population or studying male vaccination
- Articles describing HPV prevalences in non-genital infection

Of these 61 articles, 56 were original articles presenting results from one study. 5 reviews or meta-analyses presenting results were also included in the review allowing us to have a more global view of the results of the studies presented.[3–7] It should be noted that the same study was sometimes reported in several papers, up to 6 original articles not counting reviews or meta-analyses. Moreover, the same study sometimes allowed post-vs-pre as well as vaccinated-vs-unvaccinated comparisons. The main features and results of studies (reviews and meta-analyses are not presented) comparing post-vs-prevaccine and vaccinated-vs-unvaccinated prevalences are summarized in Supplementary Tables S3 and S4, respectively.

**Table S3.** Characteristics of the studies included in the systematic review (original articles only): comparison of HPV-genotype prevalences among subjects in the pre- and postvaccine eras

| **1^st^ author,**  **yr published**  **(ref)** | **Subjects:**  **country, sex, age (yr), sample source** | **Measure  of effect** | **Sub- group** | **Collection year**  **(yr postvaccine introduction)** | **Yr vaccine introduced % vaccine coverage** | **Sample size** | **HPV-genotype prevalence (%)** | | | | | | | | |
| --- | --- | --- | --- | --- | --- | --- | --- | --- | --- | --- | --- | --- | --- | --- | --- |
|  |  |  |  |  |  |  | **Vaccine** | **Nonvaccine** | | | | | | | |
| Ährlund-Richter  2019,  Grün 2016,  Ramqvist 2011  [8–10] | Sweden  Women  15–23  Clinical | Crude prevalence (2017–18: graph) |  |  | 2012 |  | 16 | 39 | 51 | | 52 | | 56 | | 59 |
|  |  |  | Pre | 2008–10 | 10.1% | 615 | 35 | 8 | 10 | | 9 | | 8 | | 8 |
|  |  |  |  | 2009–11 |  | 544 | 35 | 9 | 11 | | 10 | | 9 | | 8 |
|  |  |  | Post | 2013–15 (1–3) | 71.0% | 338 | 12.5 | 9.5 | 11 | | 10.5 | | 11 | | 14 |
|  |  |  |  | 2017–18 (5–6) | 82.1% | 178 | 8 | 15 | 20 | | 20 | | 24 | | 20 |
| Baussano 2020  [11] | Bhutan  Women  17–26  Clinical | Prevalence |  |  | 2010 |  | 6/11/16/18 | 39/45/59/68 | | | | 31/33/35/52/58 | | | |
|  |  |  | Pre | 2011–12 | 2.3% | 1130 | 8.7 | 5.0 | | | | 8.2 | | | |
|  |  |  | Post | 2018 | 66.0% | 1258 | 0.9 | 7.5 | | | | 9.5 | | | |
| Chow 2015  [12] | Australia  Women  15–24  Clinical | Crude prevalence |  |  | 2007 |  | 16/18 | 31/33/45 | | 52/58 | | | | 31/33/45/52/58 | |
|  |  |  | Pre | 2004–05 |  | 96 | **23** | 9 | | 6 | | | | 14 | |
|  |  |  |  | 2005–06 |  | 113 | **27** | 16 | | 19 | | | | 32 | |
|  |  |  |  | 2006–07 |  | 89 | **26** | 17 | | 10 | | | | 22 | |
|  |  |  | Post | 2007–08 (1) | NR | 95 | **26** | 20 | | 16 | | | | 31 | |
|  |  |  |  | 2008–09 (2) | NR | 96 | **17** | 16 | | 23 | | | | 34 | |
|  |  |  |  | 2009–10 (3) | 59% | 147 | **17** | 16 | | 20 | | | | 30 | |
|  |  |  |  | 2010–11 (4) | 57% | 129 | **18** | 12 | | 17 | | | | 26 | |
|  |  |  |  | 2011–12 (5) | 36% | 117 | **21** | 22 | | 13 | | | | 28 | |
|  |  |  |  | 2012–13 (6) | 44% | 140 | **22** | 16 | | 20 | | | | 34 | |
|  |  |  |  | 2013–14 (7) | 47% | 180 | **11** | 16 | | 20 | | | | 30 | |
| Covert 2018  Saccucci 2017  [13,14] | USA  Women  13–26  Clinical | Prevalence (adjusted propensity score) |  |  | 2007 |  | – | 39/45/59/68/70 | | 31/33/35/52/58/67 | | | | HR | |
|  |  |  | Pre | 2006–2007 |  | 371 | – | 22.1 | | 23.3 | | | | **48.6** | |
|  |  |  | Post | 2009–10 (2–3) | 59.2% | 409 |  | 31.8 | | 28.0 | | | | **58.2** | |
|  |  |  |  | 2013–14 (6–7) | 71.5% | 400 |  | 25.0 | | 16.9 | | | | **45.1** | |
|  |  |  |  | 2016–17 (9–10) | 82.5% | 360 | – | 17.3 | | 17.6 | | | | – | |

**Table S3.** Continued

| **1^st^ author,**  **yr published**  **(ref)** | **Subjects:**  **country, sex, age (yr), sample source** | **Measure  of effect** | **Sub- group** | **Collection year**  **(yr postvaccine introduction)** | **Yr vaccine introduced% vaccine coverage** | **Sample size** | **HPV-genotype prevalence (%)** | | | | | | | | |
| --- | --- | --- | --- | --- | --- | --- | --- | --- | --- | --- | --- | --- | --- | --- | --- |
|  |  |  |  |  |  |  | **Vaccine** | | **Nonvaccine** | | | | | | |
| Cummings 2012  [15] | USA  Women  14–17  Clinical | Prevalence |  |  | 2007 |  | 16/18 | |  | | | | | |  |
|  |  |  | Pre | 1999–2005 |  | 150 | **16.7** | |  | | | | | |  |
|  |  |  | Post | 2010 (5) | 89 % | 75 | **5.3** | |  | | | | | |  |
| Dillner 2018  [16] | Denmark (D), Norway (N) and Sweden (S)  Women  18–26  Clinical | Prevalence |  |  | D: 2008  N: 2009  S: 2012 |  | 16 | 18 |  | | | | | |  |
|  |  |  | Pre | 2006–2008 |  | D: 831  N: 1055  S: 986 | D: 3.5  N:16.8  S:11.8 | D: 5.3  N: 6.5  S:5.1 |  | | | | | |  |
|  |  |  | Post | 2012–2013  (D: 5–6)  (N: 4–5)  (S: 1–2) | D: 9.9–95.7%  N: 0.2–2.2%  S: 2.8–33.6% | D: 1043  N: 1011  S: 996 | D: 9.6  N:13.3  S:9.9 | D: 3.7  N: 8.2  S:3.0 |  | | | | | |  |
| Dunne  2015  Markowitz 2019  [17,18] | USA  Women  20–29  Clinical | Prevalence for all (a) and by age-subgroup*:  b: 20–24  c: 25–29 |  |  | 2007 |  | 16/18 | | HR | | 31/33/45 | | All | |  |
|  |  |  | Pre | 2007 |  | a: 4138  b:2057  c: 2081 | **a: 8.9**  **b: 10.6**  **c: 7.2** | | **a: 17.5**  **b: 20.3**  **c: 14.8** | | a: 4.3  b: 4.7  c: 3.8 | | b: 32.9  c: 24.4 | |  |
|  |  |  | Post | 2012–13 (5–6) | a: 31.9%  b: 43.7%  c: 20.5% | a: 4171  b:2057  c: 2114 | **a: 5.2**  **b: 4.5**  **c: 5.8** | | **a: 22.1**  **b: 25.6**  **c: 18.6** | | a: 3.6  b: 3.3  c: 4.0 | | b: 41.6  c: 32.8 | |  |
|  |  |  |  | 2015–16 (8–9) | b: 64.3%  c: 32.0% | b: 2059  c: 2420 | **b: 2.5**  **c: 4.6** | | **b: 25.7**  **c: 15.7** | | b: 3.5  c: 3.3 | | b: 40.6  c: 29.0 | |  |
| Feiring 2018  [19] | Norway  Women  17  General population | Prevalence |  |  | 2009 |  | 16/18 | | HR | HR except 16/18/31/33/45 | | 31/33/45/52/58 | | 31/33/45 |  |
|  |  |  | Pre | 2011 | 1.7% | 5468 | **5.1** | | **8.0** | 6.8 | | 3.9 | | 2.6 |  |
|  |  |  | Post | 2013(4) | 3.8% | 5921 | **3.2** | | **5.6** | 4.3 | | 3.0 | | 2.2 |  |
|  |  |  |  | 2014(5) | 77.0% | 6360 | **1.1** | | **4.0** | 3.6 | | 1.5 | | 0.9 |  |

**Table S3.** Continued

| **1^st^ author,**  **yr published**  **(ref)** | **Subjects:**  **country, sex, age (yr), sample source** | **Measure  of effect** | **Sub- group** | **Collection year**  **(yr postvaccine introduction)** | **Yr vaccine introduced% vaccine coverage** | **Sample size** | **HPV-genotype prevalence (%)** | | | | | | |
| --- | --- | --- | --- | --- | --- | --- | --- | --- | --- | --- | --- | --- | --- |
|  |  |  |  |  |  |  | **Vaccine** | **Nonvaccine** | | | | | |
| Guiqian 2020  [20] | China  Women  18–25  Clinical | Prevalence |  |  | 2017 |  | 16 | 52 | 58 | 39 | 33 | 68 | 51 |
|  |  |  | Pre | 2016–2017 |  | 351 | 4.27 | 5.41 | 2.56 | 1.71 | 1.99 | 1.71 | 1.42 |
|  |  |  | Post | 2018–2019 (1–2) | NA | 656 | 5.03 | 5.64 | 2.90 | 0.46 | 1.20 | 2.59 | 2.13 |
| Kahn 2012/2016  [21,22] | USA  Women  13–26  Clinical | Prevalence |  |  | 2007 |  | 16/18 | All | | | HR | | |
|  |  |  | Pre | 2006–2007 |  | 368 | **24.2** | 59.3 | | | **47.5** | | |
|  |  |  | Post | 2009–2010  (2–3) | 59.2% | 409 | **11.0** | 74.5 | | | **57.8** | | |
|  |  |  |  | 2013–2014  (6-7) | 71.3% | 400 | **8.1** | – | | | – | | |
| Kavanagh 2014/2017  Cameron 2016  [23–25] | Scotland  Women  20–21  Clinical | Prevalence |  |  | 2008 |  | 16/18 | 31/33/45 | | | 35/39/51/52/56/58/59/68 | | |
|  |  |  | Pre | 2009 | 1.26% | 1656 | **28.8** | 13.0 | | | 29.1 | | |
|  |  |  |  | 2010 | 5.77% | 1053 | **31.6** | 13.6 | | | 34.6 | | |
|  |  |  | Post | 2011 (3) | 44.6% | 1001 | **23.3** | 10.4 | | | 33.0 | | |
|  |  |  |  | 2012 (4) | 75.4% | 993 | **17.0** | 8.4 | | | 35.5 | | |
|  |  |  |  | 2013 (5) | 80.5% | 1016 | **10.1** | 6.3 | | | 33.9 | | |
|  |  |  |  | 2014 (6) | 81.2% | 1019 | **11.5** | 6.0 | | | 34.5 | | |
|  |  |  |  | 2015 (7) | 88.0% | 1610 | **4.8** | 3.0 | | | 31.4 | | |
| Machalek  2018  [26] | Australia  Women  18–24  General population | Prevalence |  |  | 2007 |  | 6/11/16/18 | 31/33/45/52/58 | | | HR | | |
|  |  |  | Pre | 2005–2007 |  | 88 | 22.7 | 14.8 | | | **33.0** | | |
|  |  |  | Post | 2010–2012  (3–5) | 87.5% | 688 | 7.3 | 19.0 | | | **36.5** | | |
|  |  |  |  | 2015  (8) | 89.5% | 200 | 1.5 | 15.5 | | | **29.5** | | |

**Table S3.** Continued

| **1^st^ author,**  **yr published**  **(ref)** | **Subjects:**  **country, sex, age (yr), sample source** | **Measure  of effect** | **Sub- group** | **Collection year**  **(yr postvaccine introduction)** | **Yr vaccine introduced% vaccine coverage** | **Sample size** | **HPV-genotype prevalence (%)** | | | |
| --- | --- | --- | --- | --- | --- | --- | --- | --- | --- | --- |
|  |  |  |  |  |  |  | **Vaccine** | | **Nonvaccine** | |
| Markowitz  2013/2016  Oliver 2017  Hirth 2019  McClung 2019  Rosenblum 2020  [1,27–31] | USA  Women  14–29  General population | Prevalence by age-subgroup*:  a: 14–19  b: 20–24  c: 25–29 |  |  | 2007 |  | 6/11/16/18 | 16/18 | 31/33/45/52/58 | HR |
|  |  |  | Pre | 2003–2006 |  | a: 1363  b: 432  c: 403 | a: 11.5  b: 18.5  c: 11.8 | **a: 7.2**  **b: 15.2**  **c: 8.1** | a: 8.4  b: 16.5  c: 10.8 | **a: 20.7**  **b: 32.9**  **c: 24.6** |
|  |  |  | Post | 2007–2010  (0–3) | a: 34.1%  b: 17.8%  c: 7.8% | a: 740  b: 445  c: 414 | a: 5.0  b: 19.9  c: 13.1 | **a: 3.6**  **b: 16.2**  **c: 10.3** | a: 6.1  b: 13.8  c: 13.1 | **a: 16.4**  **b: 37.4**  **c: 28.4** |
|  |  |  |  | 2009–2012  (2–5) | a: 51.4%  b: 32.6%  c: 14.7% | a: 736  b: 470  c: 424 | a: 4.3  b: 12.1  c: 11.7 | **a: 2.8**  **b: 10.5**  **c: 9.9** | a: 6.2  b: 12.7  c: 13.4 | **a: 18.6**  **b: 36.8**  **c: 28.1** |
|  |  |  |  | 2011–2014  (4–7) | a: 54.7%  b: 43.0%  c: 24.8% | a: 797  b: 442  c: 395 | a: 3.3  b: 7.2  c: 8.8 |  | a: 5.3  b: 13.2  c: 13.2 | **a: 15.4**  **b: 34.5**  **c: 25.5** |
|  |  |  |  | 2013–2016  (6–9) | a: 53.9%  b: 51.5%  c: 33.3% | a: 783  b: 413  c: 447 | a: 1.8  b: 5.3  c: 8.0 |  | a: 3.9  b: 13.3  c: 11.5 | – |
|  |  |  |  | 2015-2018  (8–11) | a: 54.3%  b: 59.9%  c: 40.7% | a: 666  b: 368  c: 430 | a: 1.1  b: 3.3  c: 9.1 |  | a: 2.3  b: 10.2  c: 11.6 | – |
| McGregor 2018  [32] | Australia Indigenous women  18–26  Clinical | Prevalence |  |  | 2007 |  | 6/11/16/18 | | HR | |
|  |  |  | Pre | 2005–2007 |  | 155 | 23.9 | | **32.9** | |
|  |  |  | Post | 2014–2015  (7–8) | 63% | 141 | 1.4 | | **17.0** | |

**Table S3.** Continued

| **1^st^ author,**  **yr published**  **(ref)** | **Subjects:**  **country, sex, age (yr), sample source** | **Measure  of effect** | **Sub- group** | **Collection year**  **(yr postvaccine introduction)** | **Yr vaccine introduced% vaccine coverage** | **Sample size** | **HPV-genotype prevalence (%)** | | | | | | | | | | | | | | |
| --- | --- | --- | --- | --- | --- | --- | --- | --- | --- | --- | --- | --- | --- | --- | --- | --- | --- | --- | --- | --- | --- |
|  |  |  |  |  |  |  | **Vaccine** | | **Nonvaccine** | | | | | | | | | | | | |
| Mesher  2013/2016/ 2018  [33–35] | England  Women  16–24  Clinical | Prevalence by age-subgroup*:  a: 16–18  b: 19–21  c: 22–24 |  |  | 2008 |  | 16/18 | | 31/33/45/52/58 | | | | | | | HR | | | | | |
|  |  |  | Pre | 2008 |  | a: 1047  b: 804  c: 503 | **a: 17.6**  **b: 16.9**  **c: 15.3** | | a: 14.5  b: 15.2  c: 16.7 | | | | | | | **a: 24.9**  **b: 26.9**  **c: 26.4** | | | | | |
|  |  |  | Post | 2010–2011  (2–3) | 27.3% | a: 933  b: 1463  c: 1206 | **a: 8.5**  **b: 14.2**  **c: 16.5** | | a: 16.9  b: 21.6  c: 18.4 | | | | | | | **a: 34.2**  **b: 39.1**  **c: 32.0** | | | | | |
|  |  |  |  | 2012–2013  (4–5) | 44.0% | a: 1063  b: 1310  c: 1346 | **a: 4.0**  **b: 8.7**  **c: 16.1** | | a: 14.7  b: 21.0  c: 23.3 | | | | | | | **a: 33.2**  **b: 40.2**  **c: 33.7** | | | | | |
|  |  |  |  | 2014–2015  (6–7) | 80.0% | a: 1953  b: 664  c: 120 | **a: 1.8**  **b: 2.7**  **c: 7.5** | | a: 10.2  b: 16.1  c: 18.3 | | | | | | | – | | | | | |
|  |  |  |  | 2016  (8) | 84.0% | a: 629  b: 796 | **a: 1.6**  **b: 1.6** | | a: 7.2  b: 12.7 | | | | | | | – | | | | | |
| Purriños-Hermida 2018  [36] | Spain  Women  18–26  Clinical | Prevalence |  |  | 2008 |  | 16/18 | | 31/33/45 | | | 26/35/39/51/52/53/56/58/59/66/68/73/82 | | | | | | | | | |
|  |  |  | Pre | 2008–2010 |  | 523 | **9.8** | | 5.9 | | | 13.2 | | | | | | | | | |
|  |  |  | Post | 2014–2015  (6–7) | 43% | 447 | **6.3** | | 6.3 | | | 28.2 | | | | | | | | | |
|  |  |  |  | 2016–2017  (8–9) | 53% | 298 | **3.7** | | 3.0 | | | 19.5 | | | | | | | | | |
| Söderlund-Strand  2014  [37] | Sweden  Women  13–22  Clinical | Prevalence |  |  | 2012 |  | 16 | 18 | 31 | 33 | 35 | | 39 | 45 | 51 | 52 | 56 | 58 | 59 | 66 | 68 |
|  |  |  | Pre | 2008 | 1.6–17.1% | 9644 | 14.9 | 7.9 | 7.1 | 3.7 | 1.6 | | 5.1 | 3.6 | 9.8 | 6.5 | 6.1 | 3.3 | 4.0 | 7.7 | 1.0 |
|  |  |  | Post | 2012  (0) | 17.8– 63.6% | 1433 | 8.9 | 4.7 | 7.0 | 3.1 | 1.5 | | 6.4 | 3.4 | 11.9 | 8.4 | 8.0 | 4.0 | 5.9 | 9.8 | 1.2 |
|  |  |  |  | 2013  (1) | 28.9– 77.7% | 1383 | 8.7 | 4.3 | 5.9 | 3.9 | 1.2 | | 5.6 | 4.3 | 10.6 | 9.1 | 7.3 | 3.5 | 3.7 | 9.3 | 1.0 |

**Table S3.** Continued

| **1^st^ author,**  **yr published**  **(ref)** | **Subjects:**  **country, sex, age (yr), sample source** | **Measure  of effect** | **Sub- group** | **Collection year**  **(yr postvaccine introduction)** | **Yr vaccine introduced% vaccine coverage** | **Sample size** | **HPV-genotype prevalence (%)** | | | | |
| --- | --- | --- | --- | --- | --- | --- | --- | --- | --- | --- | --- |
|  |  |  |  |  |  |  | **Vaccine** | **Nonvaccine** | | | |
| Sonnenberg 2013  [38] | England, Scotland,  Wales  Women and men  16–44  General population | Prevalence |  |  | 2008 |  | 16/ 18^b^ |  | | | |
|  |  |  | Pre | 1999–2001 |  | 684 | **11.3** |  | | | |
|  |  |  | Post | 2010–2012  (2–4) | 61.5% | 1426 | **5.8** |  | | | |
| Tabrizi  2012  2014  [39,40] | Australia  Women  18–24  Clinical | Prevalence |  |  | 2007 |  | 16/18 | 31/33/35/45 | | HR | |
|  |  |  | Pre | 2005–2007 |  | 202 | **28.7** | 10.4 | | **37.6** | |
|  |  |  | Post | 2010–2011  (3–4) | 83.7% | 404 | **6.7** | 9.2 | | **31.2** | |
|  |  |  |  | 1010–2012  (3–5) | 86.0% | 1058 | **5.4** |  | |  | |
| Woestenberg  2019  [41] | Netherlands  Women  16–24  Sexual health clinics | Prevalence |  |  | 2009 |  | 16/18 | 31 | 33 | | 45 |
|  |  |  | Pre | 2009 | 2.3% | 1110 | **22.7** | 12.4 | 4.3 | | 1.9 |
|  |  |  | Post | 2011  (2) | 6.4% | 1274 | **23.9** | 11.1 | 5.3 | | 3.6 |
|  |  |  |  | 2013  (4) | 19.2% | 1294 | **18.5** | 10.4 | 3.4 | | 3.6 |
|  |  |  |  | 2015  (6) | 36.7% | 1318 | **14.9** | 10.5 | 5.4 | | 3.2 |

HPV: human papillomavirus; HR: high risk; NV: nonvaccine genotypes; V: vaccine genotypes; Pre: prevaccine; Post: postvaccine; NR, not reported.

* Lower case letters refer age groups.

^a^ Figures available by genotype not shown here. ^b^Vaccine-genotype prevalences among 18–20-year-old women and men are reported. ^c^Nonvaccine-genotype prevalences among 18–44-year-old women and men are given.

Reviewed studies providing prevalence of NV genotypes without distinguishing them from V genotypes, were not reported in the table.

Figures in bold are those used in Figure 3 of the main text.

**Table S4.** Characteristics of the studies included in the systematic review (original articles only): comparison of HPV-genotype prevalences among vaccinated and unvaccinated subjects in the postvaccine era

| **1^st^ author,**  **yr published**  **(ref)** | **Subjects:**  **country, sex, age (yr), sample source** | **Measure  of effect** | **Collection year (yr postvaccine introduction)** | **Yr vaccine introduced% vaccine coverage** | **Sub- group** | **Sample size** | **HPV-genotype prevalence (%)** | | | | | | | | | | | | | | | | | | | |
| --- | --- | --- | --- | --- | --- | --- | --- | --- | --- | --- | --- | --- | --- | --- | --- | --- | --- | --- | --- | --- | --- | --- | --- | --- | --- | --- |
|  |  |  |  |  |  |  | **Vaccine** | | **Nonvaccine** | | | | | | | | | | | | | | | | | |
| Ährlund-Richter 2019  Grün 2016  [8,9] | Sweden  Women  15–23  Clinical | Crude prevalence (Graph for 2017-2018) |  | 2012 |  |  | 16 | | 39 | | | 51 | | | 52 | | | | | 56 | | | | 59 | | |
|  |  |  | 2013–2015  (1–3) | 71.0% | Vac+ | 240 | 5 | | 10 | | | 12 | | | 10 | | | | | 13 | | | | 12 | | |
|  |  |  |  |  | Vac– | 98 | 18 | | 9 | | | 10 | | | 13 | | | | | 9 | | | | 19 | | |
|  |  |  | 2017–2018  (5–6) | 82.1% | Vac+ | 138 | 6 | | 15 | | | 19 | | | 18 | | | | | 23 | | | | 20 | | |
|  |  |  |  |  | Vac– | 30 | 16 | | 20 | | | 27 | | | 20 | | | | | 27 | | | | 20 | | |
| Batmunkh 2020  [42] | Mongolia  Women  18–23  General | Crude prevalence |  | 2012 |  |  | 16/18/45 | | HR except 45 | | | | | | | | | | | | | | | | | |
|  |  |  | 2017–2018  (5–6) | 47.3% | Vac+ | 726 | 4.8 | | 32.4 | | | | | | | | | | | | | | | | | |
|  |  |  |  |  | Vac– | 790 | 17.2 | | 24.6 | | | | | | | | | | | | | | | | | |
| Baussano 2020  [11] | Bhutan  Women  17–29  Clinical | Prevalence |  | 2010 |  |  |  | | All | | | | | | | | | | | | | | | | | |
|  |  |  | 2018  (8) | 66.0% | Vac+ | 1048 |  | | 33.5 | | | | | | | | | | | | | | | | | |
|  |  |  |  |  | Vac– | 343 |  | | 29.7 | | | | | | | | | | | | | | | | | |
| Bereson 2017  [43] | USA  Women  18–26  General | Crude prevalence (graph) |  | 2007 |  |  | 16/18 | | 31/33/35/39/45/51/52/53/56/58/59/66/68/73/82 | | | | | | | | | | | | | | | | | |
|  |  |  | 2009–2010  (2–3) | 7.4% | Vac+ | 25 | **3** | | 48 | | | | | | | | | | | | | | | | | |
|  |  |  |  |  | Vac– | 328 | **18** | | 40 | | | | | | | | | | | | | | | | | |
|  |  |  | 2011–2012  (4–5) | 10.1% | Vac+ | 40 | **3** | | 38 | | | | | | | | | | | | | | | | | |
|  |  |  |  |  | Vac– | 254 | **9** | | 40 | | | | | | | | | | | | | | | | | |
|  |  |  | 2013–2014  (6–7) | 13.8% | Vac+ | 63 | **2** | | 38 | | | | | | | | | | | | | | | | | |
|  |  |  |  |  | Vac– | 245 | **8** | | 30 | | | | | | | | | | | | | | | | | |
| Brogly 2014  [44] | Boston, MA, USA  Women  21–30  Clinical high risk | Crude prevalence |  | 2007 |  |  | 16 | 18 | 45 | | 53 | | | | | | 59 | | | | | 66 | | | | |
|  |  |  | 2011–2012  (4–5) | 41% | Vac+ | 96 | 2.1 | 2.9 | 3.25 | | 3.25 | | | | | | 1.0 | | | | | 2.1 | | | | |
|  |  |  |  |  | Vac– | 136 | 2.2 | 0 | 1.5 | | 1.5 | | | | | | 0.8 | | | | | 1.5 | | | | |
| Carozzi 2018  [45] | Italy  Women  18–30  General | Crude prevalence |  | 2007 |  |  | 16/18 | | HR | HR but 31 | | | HR but 31/33/45 | 31/33/45 | | 31 | | 39 | 51 | | 52 | | 56 | | 58 | 59 |
|  |  |  | 2012–2014  (5–7) | 59.0% | Vac+ | 771 | **0.6** | | **8.8** | 8.3 | | | 8.2 | 1.5 | | 1.3 | | 1.8 | 2.9 | | 2.1 | | 1.0 | | 1.3 | 0.5 |
|  |  |  |  |  | Vac– | 537 | **5.2** | | **8.6** | 6.9 | | | 6.3 | 2.7 | | 2.1 | | 0.9 | 1.3 | | 2.2 | | 0.9 | | 0.9 | 0.7 |

**Table S4.** Continued

| **1^st^ author,**  **yr published**  **(ref)** | **Subjects:**  **country, sex, age (yr), sample source** | **Measure  of effect** | **Collection year (yr postvaccine introduction)** | **Yr vaccine introduced% vaccine coverage** | **Sub- group** | **Sample size** | **HPV-genotype prevalence (%)** | | | |
| --- | --- | --- | --- | --- | --- | --- | --- | --- | --- | --- |
|  |  |  |  |  |  |  | **Vaccine** | **Nonvaccine** | | |
| Covert 2018  Saccucci 2017  [13,14] | USA  Women  13–26  Clinical | Prevalence |  | 2007 |  |  |  | 39/45/59/68/70 | 31/33/35/52/58/67 | HR |
|  |  |  | 2009–2010  (2–3) | 59.2% | Vac+ | 242 |  | 36.9 | 28.9 | **63.9** |
|  |  |  |  |  | Vac– | 167 |  | 23.8 | 29.1 | **47.9** |
|  |  |  | 2013–2014  (6–7) | 71.5% | Vac+ | 286 |  | 23.3 | 15.5 | **41.8** |
|  |  |  |  |  | Vac– | 114 |  | 28.6 | 27.7 | **56.7** |
|  |  |  | 2016–2017  (9–10) | 82.5% | Vac+ | 297 |  | 20.0 | 12.3 |  |
|  |  |  |  |  | Vac– | 63 |  | 6.3 | 35.3 |  |
| Dunne 2015  Markowitz 2019  [17,18] | USA  Women  20–29  Clinical | Prevalence by age-subgroup*:  a: 20–24  b: 25–29 |  | 2007 |  |  | 16/18 | All | HR | 31/33/45 |
|  |  |  | 2012–2013  (5–6) | a: 43.7%  b: 20.5% | Vac+ | a: 898  b: 433 | **a: 1.7**  **b: 4.6** | a: 41.9  b: 41.6 | **a: 25.8**  **b: 21.7** | a: 3.3  b: 4.4 |
|  |  |  |  |  | Vac– | a: 1159  b: 1681 | **a: 6.7**  **b: 6.1** | a: 41.3  b: 30.6 | **a: 25.5**  **b: 17.8** | a: 3.2  b: 3.9 |
|  |  |  | 2015–2016  (8–9) | a: 64.3%  b: 32.0% | Vac+ | a: 1323  b: 775 | **a: 1.1**  **b:2.5** | a: 42.3  b: 33.7 | **a: 26.8**  **b: 19.1** | a: 3.3  b: 3.6 |
|  |  |  |  |  | Vac– | a:736  b: 1645 | **a: 5.0**  **b: 5.6** | a: 37.5  b: 26.8 | **a: 23.9**  **b: 14.2** | a: 3.9  b: 3.1 |
| Enerly 2019  [46] | Norway  Women  18–20  General (Facebook) | Crude prevalence |  | 2009 |  |  | 16/18 | All | | |
|  |  |  | 2016–2017  (7–8) | 76.6% | Vac+ | 239 | **0.4** | 38.5 | | |
|  |  |  |  |  | Vac– | 73 | **4.1** | 38.4 | | |
| Franceschi 2016  [47] | Bhutan  Rwanda  Women  17–22  General population | Prevalence (urine sample  method with  44 HPV detectable) |  |  |  |  | 6/11/16/18 | 31/33/35/39/45/52/58/59/68/70 | | |
|  |  |  | Bhutan: 2013 (3) | 2010 92.0% | Vac+ | 896 | 1.2 | 6.7 | | |
|  |  |  |  |  | Vac– | 77 | 1.3 | 6.5 | | |
|  |  |  | Rwanda: 2013–2014 (2–3) | 2011 43.1% | Vac+ | 393 | 2.8 | 8.1 | | |
|  |  |  |  |  | Vac– | 519 | 6.4 | 12.3 | | |
| Goggin 2017  [48] | Canada  Women  17–29  General population | Crude prevalence by age group  a: 17–19  b: 20–22  c: 23–29 |  | 2008 |  |  | 6/11/16/18 |  | | |
|  |  |  | 3/2013–7/2014  (5–6) | 62.3%  a: 83.5%  b: 65.7%  c: 19.1% | Vac+ | 511 | a: 0.3  b: 1.4  c: 10.5 |  | | |
|  |  |  |  |  | Vac– | 1039 | a: 8.2  b: 9.9  c: 11.9 |  | | |

**Table S4.** Continued

| **1^st^ author,**  **yr published**  **(ref)** | **Subjects:**  **country, sex, age (yr), sample source** | **Measure  of effect** | **Collection year (yr postvaccine introduction)** | **Yr vaccine introduced% vaccine coverage** | **Sub- group** | **Sample size** | **HPV-genotype prevalence (%)** | | | | | | | | | | | | | | | | | | | | | | | | | |
| --- | --- | --- | --- | --- | --- | --- | --- | --- | --- | --- | --- | --- | --- | --- | --- | --- | --- | --- | --- | --- | --- | --- | --- | --- | --- | --- | --- | --- | --- | --- | --- | --- |
|  |  |  |  |  |  |  | **Vaccine** | | **Nonvaccine** | | | | | | | | | | | | | | | | | | | | | | | |
| Gray  2018  2019  [49,50] | Finland  Women  18.5–19  Community randomized trial | Adjusted prevalence |  | 2007 |  |  | 16 | 18 | 31 | | 33 | | 35 | | 39 | | 45 | | | 51 | | 52 | | 56 | | 58 | | 59 | | 66 | |  |
|  |  |  | 2010–2012  (3–5)  (born: 1992–1993) | 50% women | Vac+ | Arm A: 2929 (total) | 0.6 | 0.2 | 0.7 | | 2.0 | | 0.7 | | 3.5 | | 0.5 | | | 8.0 | | 4.1 | | 3.3 | | 2.6 | | 2.8 | | 4.4 | |  |
|  |  |  |  |  |  | Arm B: 3059 (total) | 0.6 | 0.3 | 0.9 | | 1.6 | | 1.2 | | 3.1 | | 0.5 | | | 8.1 | | 4.6 | | 4.7 | | 2.0 | | 3.0 | | 4.6 | |  |
|  |  |  |  |  | Vac– | 3375 (total) | 7.1 | 3.9 | 3.2 | | 2.6 | | 1.2 | | 2.7 | | 2.1 | | | 5.2 | | 4.2 | | 3.9 | | 2.8 | | 2.4 | | 3.1 | |  |
|  |  |  | 2012–2014  (5–7)  (born: 1994–1995) |  | Vac+ | Arm A | 0.3 | 0.1 | 0.8 | | 1.0 | | 0.5 | | 2.8 | | 0.3 | | | 6.2 | | 3.5 | | 5.2 | | 2.8 | | 2.9 | | 3.4 | |  |
|  |  |  |  |  |  | Arm B | 0.6 | 0.2 | 0.7 | | 1.1 | | 0.9 | | 2.2 | | 0.3 | | | 6.4 | | 3.3 | | 4.2 | | 2.1 | | 2.7 | | 3.9 | |  |
|  |  |  |  |  | Vac– |  | 7.0 | 4.0 | 3.7 | | 2.4 | | 1.5 | | 3.0 | | 1.9 | | | 7.0 | | 4.4 | | 5.1 | | 2.1 | | 2.7 | | 4.2 | |  |
| Grün 2015  [51] | Sweden  Women  15–23  Clinical | Crude prevalence |  | 2007–2012 |  |  | 16 | 18 | 31 | 33 | | 35 | | 39 | | 45 | | 51 | | | 52 | | 56 | | 58 | | 59 | | 66 | | 68 |  |
|  |  |  | 2013–2014  (1–7) | 73%  (vaccination after sexual debut) | Vac+ | 154 | 5 | 2 | 1 | 7 | | 1 | | 9 | | 5 | | 13 | | | 9 | | 11 | | 1 | | 11 | | 11 | | 4 |  |
|  |  |  |  |  | Vac– | 57 | 22 | 5 | 8 | 5 | | 3 | | 10 | | 8 | | 10 | | | 8 | | 10 | | 5 | | 8 | | 15 | | 3 |  |
| Guo 2015  [52] | USA  Women  20–26  General population | Crude prevalence |  | 2007 |  |  | 16/18 | | All | | | | | | | | | | HR | | | | | | | | | | | | | |
|  |  |  | 2007–2012  (0–5) | 21.4% | Vac+ | 177 | **7.1** | | 62.4 | | | | | | | | | | **52.1** | | | | | | | | | | | | | |
|  |  |  |  |  | Vac– | 701 | **13.9** | | 52.8 | | | | | | | | | | **40.4** | | | | | | | | | | | | | |
| INSP Québec  2016  [53] | Canada  Women  17–29  General population | Crude prevalence |  | 2007 |  |  | 6/11/16/18 | | 31/33/45 | | | | | | | | | | HR | | | | | | | | | | | | | |
|  |  |  | 3/2013–7/2014  (6–7) | 62.3% | Vac+ | 1039 | 1.5 | | 3.5 | | | | | | | | | | **20.5** | | | | | | | | | | | | | |
|  |  |  |  |  | Vac– | 511 | 11.0 | | 5.7 | | | | | | | | | | **19.4** | | | | | | | | | | | | | |

**Table S4.** Continued

| **1^st^ author,**  **yr published**  **(ref)** | **Subjects:**  **country, sex, age (yr), sample source** | **Measure  of effect** | **Collection year (yr postvaccine introduction)** | **Yr vaccine introduced% vaccine coverage** | **Sub- group** | **Sample size** | **HPV-genotype prevalence (%)** | | | | | | | | | | | | | | | |
| --- | --- | --- | --- | --- | --- | --- | --- | --- | --- | --- | --- | --- | --- | --- | --- | --- | --- | --- | --- | --- | --- | --- |
|  |  |  |  |  |  |  | **Vaccine** | **Nonvaccine** | | | | | | | | | | | | | | |
| Jeannot 2018  [54] | Switzerland  Women  18–31  University | Prevalence |  | 2008 |  |  | 16/18 | HR | | | | | | | | | | | | | | |
|  |  |  | 1/2016–10/2017  (8–9) | 69% | Vac+ | 284 | **1.1** | **10.3** | | | | | | | | | | | | | | |
|  |  |  |  |  | Vac– | 125 | **7.2** | **11.2** | | | | | | | | | | | | | | |
| Kahn  2012  2016  [21,22] | USA  Women  13–26  Clinical | Prevalence |  | 2006–2007 |  |  | 16/18 | All | | | | | | HR | | | | | | | | |
|  |  |  | 2009–2010  (3–4) | 59.2% | Vac+ | 242 | **7.9** | 77.6 | | | | | | **65.2** | | | | | | | | |
|  |  |  |  |  | Vac– | 167 | **15.6** | 70.1 | | | | | | **47.3** | | | | | | | | |
|  |  |  | 2013–2014  (4–8) | 71.3% | Vac+ | 286 | **3.9** |  | | | | | |  | | | | | | | | |
|  |  |  |  |  | Vac– | 114 | **18.4** |  | | | | | |  | | | | | | | | |
| Kavanagh 2014  2017  Cameron 2016  [23–25] | Scotland  Women  20–21  Clinical | Prevalence |  | 2008 |  |  | 16/18 | 31/33/45 | | | | | | 35/39/51/52/56/58/59/68 | | | | | | | | |
|  |  |  | 2009–2012  (1–4) | 24.3% | Vac+ | 1100 | **13.6** | 6.8 | | | | | | 31.6 | | | | | | | | |
|  |  |  |  |  | Vac– | 3418 | **29.8** | 13.1 | | | | | | 32.4 | | | | | | | | |
|  |  |  | 2009–2013  (1–5) | 33.9% | Vac+ | 1853 | **11.0** | 6.2 | | | | | | 32.9 | | | | | | | | |
|  |  |  |  |  | Vac– | 3619 | **29.4** | 12.9 | | | | | | 32.5 | | | | | | | | |
|  |  |  | 2009–2015  (1–7) | 49.7% | Vac+ | 3962 | **8.1** | 4.5 | | | | | | 32.9 | | | | | | | | |
|  |  |  |  |  | Vac– | 4008 | **27.8** | 12.6 | | | | | | 32.4 | | | | | | | | |
| Kumakech 2016  [55] | Uganda  Women  15–24  General population | Prevalence |  | 2008–2009 |  |  | 16/18 | all | 31 | 33 | 35 | 39 | 45 | | 51 | 52 | 56 | 58 | 59 | 66 | 68 |  |
|  |  |  | 7/2014–8/2014  (4–5) | 51.1% | Vac+ | 205 | **0.5** | 29.3 | 1.0 | 0.5 | 0.5 | 1.5 | 0.5 | | 2.4 | 3.9 | 0.5 | 3.9 | 3.9 | 2.4 | 0.5 |  |
|  |  |  |  |  | Vac– | 196 | **5.6** | 35.7 | 2.6 | 0 | 2.1 | 1.0 | 0.5 | | 4.1 | 5.6 | 2.1 | 7.2 | 2.1 | 4.6 | 1.5 |  |
| Lynge  2020  [56] | Denmark  Women  23–24  General population | Prevalence |  | 2009 |  |  | 16/18 | HR | | | | | | | | | | | | | | |
|  |  |  | 02/2017-04/2019  (8–10) | 92% | Vac+ | 5685 | **0.4** | **34.8** | | | | | | | | | | | | | | |
|  |  |  |  |  | Vac- | 518 | **6.6** | **30.1** | | | | | | | | | | | | | | |
| Machalek  2018  [26] | Australia  Women  18–24  Clinical | Prevalence |  | 2007 |  |  | 6/11/16/18 | 31/33/45/52/58 | | | | HR | | | | | | | | | | |
|  |  |  | 2015  (8) | 89.5% | Vac+ | 203 | 1.0 | 14.3 | | | | **27.6** | | | | | | | | | | |
|  |  |  |  |  | Vac– | 54 | 1.9 | 13.0 | | | | **14.8** | | | | | | | | | | |

**Table S4.** Continued

| **1^st^ author,**  **yr published**  **(ref)** | **Subjects:**  **country, sex, age (yr), sample source** | **Measure  of effect** | **Collection year (yr postvaccine introduction)** | **Yr vaccine introduced% vaccine coverage** | **Sub- group** | **Sample size** | **HPV-genotype prevalence (%)** | | | | | | | | | | | | | | | | | | | | | | |
| --- | --- | --- | --- | --- | --- | --- | --- | --- | --- | --- | --- | --- | --- | --- | --- | --- | --- | --- | --- | --- | --- | --- | --- | --- | --- | --- | --- | --- | --- |
|  |  |  |  |  |  |  | **Vaccine** | | **Nonvaccine** | | | | | | | | | | | | | | | | | | | | |
| Markowitz  2013/2016  Oliver 2017  Hirth 2019  McClung 2019  Rosenblum 2020  [1,27–31] | USA  Women  14–24  (age subgroup by data collection)  General population | Prevalence |  | End of 2006 |  |  | 6/11/16/18 | | | HR | | | | | | | | | | 31/33/45 | | | | | | | | | |
|  |  |  | 2007–2010  (1–4)  age 14–19 yr | 68.3% | Vac+ | 239 | 3.1 | | | **35.2** | | | | | | | | | |  | | | | | | | | | |
|  |  |  |  |  | Vac– | 111 | 12.6 | | | **25.3** | | | | | | | | | |  | | | | | | | | | |
|  |  |  | 2009–2012  (3–6)  age 14–24 yr | 49.1% | Vac+ | 347 | 2.0 | | | **34.2** | | | | | | | | | | 4.9 | | | | | | | | | |
|  |  |  |  |  | Vac– | 360 | 12.2 | | | **36.7** | | | | | | | | | | 5.0 | | | | | | | | | |
|  |  |  | 2011–2014  (5–8)  age 14–24 yr | 39.5% | Vac+ | 287 | 2.1 | | | **37.2** | | | | | | | | | | 4.9 | | | | | | | | | |
|  |  |  |  |  | Vac– | 439 | 16.9 | | | **39.1** | | | | | | | | | | 5.8 | | | | | | | | | |
| McGregor 2018  [32] | Australia  Indigenous women  18–26  Clinical | Prevalence |  | 2007 |  |  | 6/11/16/18 | | | HR | | | | | | | | | | | | | | | | | | | |
|  |  |  | 2014–2015  (7–8) | 63% | Vac+ | 118 | 0.9 | | | **17.0** | | | | | | | | | | | | | | | | | | | |
|  |  |  |  |  | Vac– | 21 | 4.8 | | | **19.1** | | | | | | | | | | | | | | | | | | | |
| Mesher 2018  [35] | England, UK  Women  16–24  Clinical | Prevalence by age subgroup A: Vac ≤15 yr  B: Older catch-up |  | 2008 |  |  | 16/18 | | | 31/33/45 | | | | | | | | | | | 31/33/45/52/58 | | | | | | | | |
|  |  |  | 2010–2016  (2–8) | 86.7% | Vac+ | A: 1176  B: 614 | **A: 1.4**  **B: 6.2** | | | A: 3.6  B: 5.7 | | | | | | | | | | | A: 11.7  B: 18.9 | | | | | | | | |
|  |  |  |  |  | Vac– | A: 117  B: 289 | **A: 8.5**  **B: 13.5** | | | A: 7.7  B: 9.7 | | | | | | | | | | | A: 15.4  B: 24.2 | | | | | | | | |
| Murall 2020  [57] | France  Women  18–25  clinical | Prevalence (graph) |  | 2007 |  |  | 16 | 18 | | 31 | 33 | | 35 | | | 39 | | 45 | | | 51 | | 52 | | 56 | | 58 | | 59 |
|  |  |  | 2019  (12) | 49% | Vac+ | 73 | 2.7 | 5.5 | | 2.7 | 0 | | 3.9 | | | 3.9 | | 0 | | | 3.9 | | 12.5 | | 1.4 | | 1.6 | | 3.9 |
|  |  |  |  |  | Vac– | 76 | 14.8 | 2.3 | | 14.8 | 0 | | 9.4 | | | 6.6 | | 2.4 | | | 24.1 | | 9.4 | | 10.9 | | 1.5 | | 1.5 |
| Purriños-Hermida  2018  [36] | Spain  Women  18–26  Clinical | Prevalence |  | 2008 |  |  | 16/18 | | | 31/33/45 | | | | | All except 31/33/45 | | | | | | | | | | | | | | |
|  |  |  | 2014–2017  (6–9) | 47% | Vac+ | 392 | **0.8** | | | 1.1 | | | | | 24.6 | | | | | | | | | | | | | | |
|  |  |  |  |  | Vac– | 353 | **9.2** | | | 8.4 | | | | | 24.7 | | | | | | | | | | | | | | |
| Schlecht 2012  [58] | NY, USA  Women  12–19  Clinical HR | Prevalence (graph) |  | 2007 |  |  | 16 | 18 | | 31 | | 33 | | 35 | | | 39 | | 45 | | | 52 | | 58 | | 59 | | 68 | |
|  |  |  | 2010  (3) | 79.5% (50.7% with 3 doses) | Vac+ | 327 | 2.1 | 0.8 | | 1.9 | | 0.9 | | 2.1 | | | 3.1 | | 0.6 | | | 5.9 | | 5.3 | | 1.9 | | 2.8 | |
|  |  |  |  |  | Vac– | 137 | 6.1 | 3.9 | | 4.6 | | 0.8 | | 1.5 | | | 3.9 | | 3.1 | | | 3.9 | | 4.6 | | 3.9 | | 2.3 | |

**Table S4.** Continued

| **1^st^ author,**  **yr published**  **(ref)** | **Subjects:**  **country, sex, age (yr), sample source** | **Measure  of effect** | **Collection year (yr postvaccine introduction)** | **Yr vaccine introduced% vaccine coverage** | **Sub- group** | **Sample size** | **HPV-genotype prevalence (%)** | | | | | | | | | | | | | | | | | |
| --- | --- | --- | --- | --- | --- | --- | --- | --- | --- | --- | --- | --- | --- | --- | --- | --- | --- | --- | --- | --- | --- | --- | --- | --- |
|  |  |  |  |  |  |  | **Vaccine** | | **Nonvaccine** | | | | | | | | | | | | | | | |
| Shilling 2021  [59] | Australia  Women  18–35  Clinical | Prevalence |  | April 2007 |  |  | 6/11/16/18 | | All except 31/33/45 | | | | | | | | | 31/33/45 | | | | | | |
|  |  |  | 2015–2018  (8–11) | 77.7% | Vac+ | 1216 | 2.0 | | 40.2 | | | | | | | | | 3.8 | | | | | | |
|  |  |  |  |  | Vac– | 348 | 5.5 | | 34.8 | | | | | | | | | 6.6 | | | | | | |
| Tabrizi  2012  2014  [39,40] | Australia  Women  18–24  Clinical | Prevalence |  | April 2007 |  |  | 6/11/16/18 | | HR | | | | | | | | | 31/33/35/45 | | | | | | |
|  |  |  | 2010–2011  (3–4) | 83.7% | Vac+ | 338 | 5.0 | | **30.8** | | | | | | | | | 7.7 | | | | | | |
|  |  |  |  |  | Vac– | 57 | 15.8 | | **35.1** | | | | | | | | | 15.8 | | | | | | |
|  |  |  | 2010–2012  (3–5) | 86.0% | Vac+ | 518 | 2 | | **34** | | | | | | | | | 6 | | | | | | |
|  |  |  |  |  | Vac– | 149 | 19 | | **39** | | | | | | | | | 15 | | | | | | |
| Wendland 2021  [60] | Brazil  Women  16–25  General population | Prevalence |  | 2014 |  |  | 6/11/16/18 | | HR | | | | | | | | | | | | | | | |
|  |  |  | 09/2016–11/2017  (2–3) |  | Vac+ | 677 | 6.76 | | **40.47** | | | | | | | | | | | | | | | |
|  |  |  |  | 11.9% | Vac– | 5268 | 15.64 | | **32.63** | | | | | | | | | | | | | | | |
| Woestenberg  2018  [61] | Netherlands Women  16–24  Sexual health clinics | Prevalence (graph) |  | 2009 |  |  | 16 | 18 | 31 | 33 | | 35 | | 39 | 45 | | 51 | | 52 | | 56 | | 58 | 59 |
|  |  |  | 2011-2015  (2-6) | 59.7% | Vac+ | 649 | 0.9 | 0.9 | 3.4 | 2.3 | | 1.1 | | 6.0 | 0.5 | | 24.0 | | 12.0 | | 10.3 | | 2.4 | 5.7 |
|  |  |  |  |  | Vac– | 438 | 12 | 6.6 | 7.1 | 3.1 | | 3.1 | | 7.4 | 3.1 | | 24.6 | | 17.7 | | 8.3 | | 3.4 | 3.1 |
| Wright 2019  [62] | USA  Women  21–34  Clinical | Prevalence  by age subgroup*:  a: 21–24  b: 25–29  c: 30–34 |  | End 2006–2007 |  |  | 16/18 | | 31 | | 33/58 | | 35/39/68 | | | 56/59/66 | | | | HR | | 45/51/52/35/39/56/59/66/68 | | |
|  |  |  | 8/26/2013–6/12/2015  (6–8) | a: 37.4%  b: 22.4%  c: 7.4% | Vac+ | a: 1424  b: 1187  c: 366 | **a: 1.3**  **b: 2.5**  **c: 2.5** | | a: 1.5  b: 1.0  c: 1.9 | | a: 3.0  b: 1.5  c: 0 | | a: 7.8  b: 5.5  c: 3.6 | | | a: 10.8  b: 6.1  c: 4.6 | | | | **a: 25.7**  **b: 17.4**  **c: 10.1** | | a: 21.6  b: 14.8  c: 8.2 | | |
|  |  |  |  |  | Vac– | a: 2380  b:4118  c: 4678 | **a: 7.7**  **b: 6.4**  **c: 4.2** | | a: 2.6  b: 2.4  c: 2.1 | | a: 3.4  b: 2.6  c: 1.7 | | a: 7.9  b: 5.2  c: 3.5 | | | a: 9.0  b: 5.2  c:3.7 | | | | **a: 24.0**  **b: 16.7**  **c: 11.9** | | a: 19.1  b: 12.4  c: 8.4 | | |

HPV: human papillomavirus; HR: high risk; NV: nonvaccine genotypes; V: vaccine genotypes; Vac+: vaccinated; Vac–: unvaccinated; INSP: Institut National de Santé Publique.

* Lower case letters refer to age groups.

Reviewed studies providing prevalence of NV genotypes without distinguishing them from V genotypes, were not reported in the table.

Figures in bold are those used in Figure 3 of the main text.

## Sensitivity analysis of the impact of strength of interaction

For ease of comparison, Figure S2 (top panel) displays absolute prevalence-difference values, |$\Delta_{prev}|$, at various times after vaccine introduction. Results are shown for NV genotypes, with positive differences for competitive scenarios (γ < 1) and negative differences for synergistic scenarios (γ > 1). Results are not shown for V genotypes, whose differences were similar, regardless of the interaction strength applied.

| 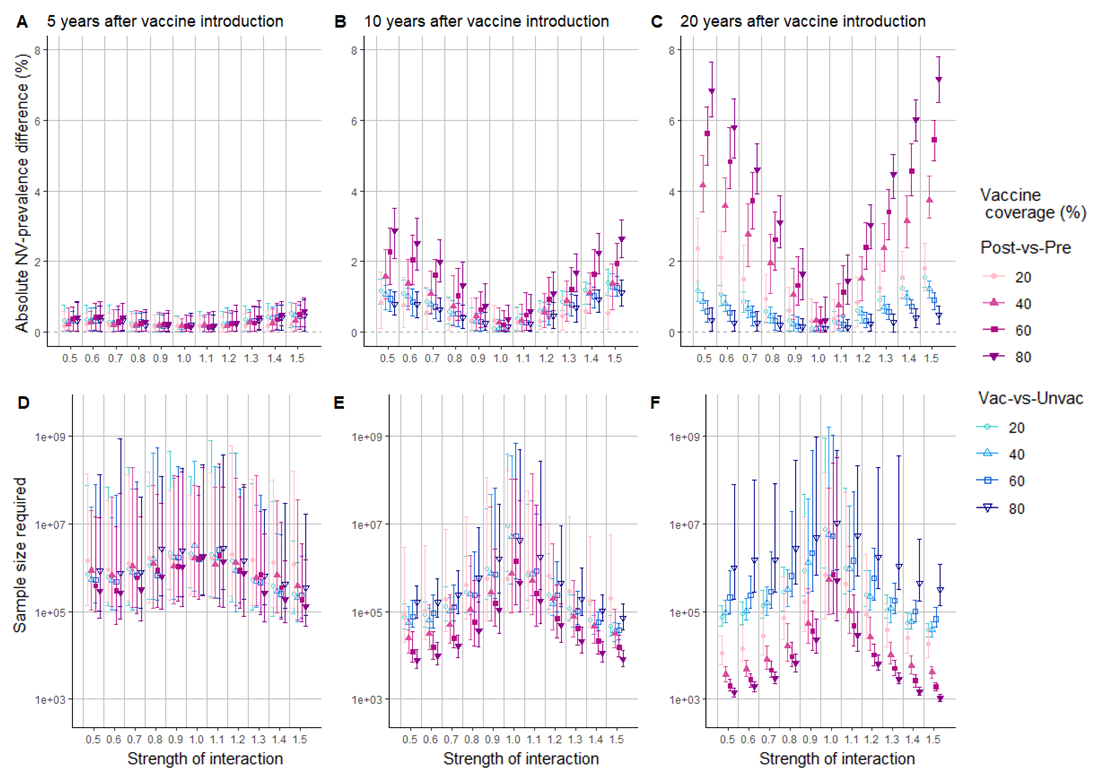 |
| --- |
| **Figure S2.** NV-genotype–prevalence differences (A–C in absolute values) and corresponding sample sizes (D–F) according to interaction strength at 5 (A & D), 10 (B & E) or 20 years (C & F) after vaccine introduction. Results shown are medians and 90% empirical intervals over 100 simulations. For synergistic values, prevalence differences are negative; they are presented here as absolute values to facilitate comparability. |

## Minimum sample size of an existing prevaccine study

Through the postvaccine prevalence *P*_1_, the prevaccine sample size depends on immunization coverage and time since vaccine introduction (Figure S3). With 40% vaccine coverage, detection of prevalence differences 20 years after vaccine introduction would require a median prevaccine sample of 12,531 individuals under the weakest interaction-strength scenario. Under the stronger competitive interaction, this required sample size is reduced to values lower than 1730 women.

| 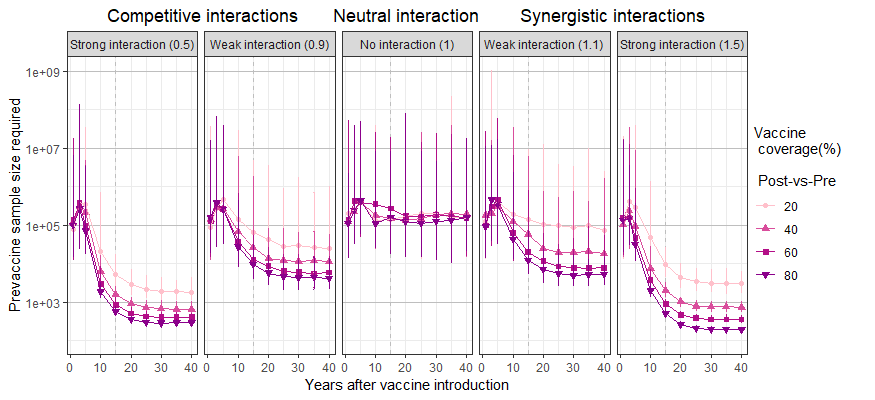 |
| --- |
| **Figure S3.** Sample size in prevaccine era for post-vs-prevaccine design according to time since vaccine introduction, strength of interaction and vaccine coverage. The dashed vertical line at 15 years indicates when all age cohorts have been offered the vaccine. Results shown are median values and 90% empirical intervals over 100 simulations. |

## Analyzing the dynamics according to sexual activity for a single NV genotype

| 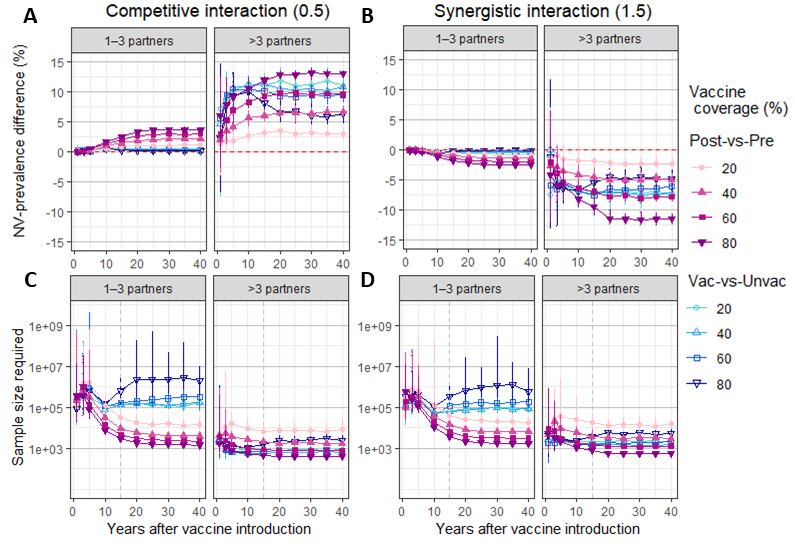 |
| --- |
| **Figure S4.** NV-genotype–prevalence difference (A, B) and corresponding sample size (C, D) over time in the case of strong competitive (0.5; A, C) and synergistic (1.5; B, D) interactions (γ) according to the individual’s number of partners during the past year, the epidemiological study design (post-vs-pre or vaccinated-vs-unvaccinated), and vaccine coverage. The dashed vertical line at 15 years indicates when all age cohorts have been offered the vaccine. The results shown are medians and 90% empirical intervals over 100 simulations. |

In the single NV-genotype analysis, the NV-prevalence difference was more pronounced among individuals with >3 partners than among those with 1–3 partners, regardless of the epidemiological study design and vaccine coverage (Figure S4A and B).

Prevalence differences among individuals with >3 partners were observed earlier for the vaccinated-vs-unvaccinated comparison and remained more pronounced for vaccine coverage ≤40% 15 years after vaccine introduction. Interestingly, required sample sizes were smaller, compared to the analysis for individuals with 1–3 partners or the general population, especially for the vaccinated-vs-unvaccinated comparison, which required a very large number of subjects to show a statistically significant prevalence difference (Figure S4C and D). For example, for 60% vaccine coverage 15 years after vaccine introduction, the median sample sizes for individuals with >3 partners were, respectively for competitive and synergistic interactions, 1,518 and 875 women for the post-vs-prevaccine comparison (total sample size of 9,130 and 6,277 women) and 1,402 and 682 women for the vaccinated-vs-unvaccinated comparison (total sample size of 64,039 and 69,709 women).

# References

1. **Markowitz LE, *et al.*** Reduction in human papillomavirus (HPV) prevalence among young women following HPV vaccine introduction in the United States, National Health and Nutrition Examination Surveys, 2003-2010. *The Journal of Infectious Diseases* 2013; **208**: 385–393.

2. **Breslow NE, Day NE**. Statistical methods in cancer research. Volume II--The design and analysis of cohort studies. *IARC scientific publications* 1987; : 1–406.

3. **Du J, *et al.*** Human papilloma virus (HPV) prevalence upon HPV vaccination in Swedish youth: a review based on our findings 2008-2018, and perspectives on cancer prevention. *Archives of Gynecology and Obstetrics* 2021; **303**: 329–335.

4. **Drolet M, *et al.*** Population-level impact and herd effects following human papillomavirus vaccination programmes: a systematic review and meta-analysis. *The Lancet Infectious Diseases* 2015; **15**: 565–580.

5. **Malagón T, *et al.*** Cross-protective efficacy of two human papillomavirus vaccines: a systematic review and meta-analysis. *The Lancet Infectious Diseases* 2012; **12**: 781–789.

6. **Mesher D, *et al.*** Population-Level Effects of Human Papillomavirus Vaccination Programs on Infections with Nonvaccine Genotypes. *Emerging Infectious Diseases* 2016; **22**: 1732–1740.

7. **Steben M, *et al.*** A review of the impact and effectiveness of the quadrivalent human papillomavirus vaccine: 10 years of clinical experience in canada. *Journal of obstetrics and gynaecology Canada: JOGC = Journal d’obstetrique et gynecologie du Canada: JOGC* 2018; **40**: 1635–1645.

8. **Ährlund-Richter A, *et al.*** Changes in cervical human papillomavirus (HPV) prevalence at a youth clinic in Stockholm, sweden, a decade after the introduction of the HPV vaccine. *Frontiers in Cellular and Infection Microbiology* 2019; **9**: 59.

9. **Grün N, *et al.*** Follow-up on oral and cervical human papillomavirus prevalence 2013-2015 in youth at a youth clinic in Stockholm, Sweden. *Infectious Diseases (London, England)* 2016; **48**: 169–170.

10. **Ramqvist T, *et al.*** Pre-vaccination prevalence of human papillomavirus types in the genital tract of 15-23-year-old women attending a youth health clinic in Stockholm, Sweden. *Scandinavian Journal of Infectious Diseases* 2011; **43**: 115–121.

11. **Baussano I, *et al.*** Prevalence of Human Papillomavirus and Estimation of Human Papillomavirus Vaccine Effectiveness in Thimphu, Bhutan, in 2011-2012 and 2018 : A Cross-sectional Study. *Annals of Internal Medicine* 2020; **173**: 888–894.

12. **Chow EPF, *et al.*** Human papillomavirus in young women with Chlamydia trachomatis infection 7 years after the Australian human papillomavirus vaccination programme: a cross-sectional study. *The Lancet. Infectious Diseases* 2015; **15**: 1314–1323.

13. **Covert C, *et al.*** Evidence for cross-protection but not type-replacement over the 11 years after human papillomavirus vaccine introduction. *Human Vaccines & Immunotherapeutics* Taylor & Francis, 2019; **15**: 1962–1969.

14. **Saccucci M, *et al.*** Non-vaccine-type human papillomavirus prevalence after vaccine introduction: no evidence for type replacement but evidence for cross-protection. *Sexually Transmitted Diseases* 2018; **45**: 260–265.

15. **Cummings T, *et al.*** Reduction of HPV infections through vaccination among at-risk urban adolescents. *Vaccine* 2012; **30**: 5496–5499.

16. **Dillner J, *et al.*** Decline of HPV infections in Scandinavian cervical screening populations after introduction of HPV vaccination programs. *Vaccine* 2018; **36**: 3820–3829.

17. **Dunne EF, *et al.*** Reduction in human papillomavirus vaccine type prevalence among young women screened for cervical cancer in an integrated US healthcare delivery system in 2007 and 2012–2013. *The Journal of Infectious Diseases* Oxford Academic, 2015; **212**: 1970–1975.

18. **Markowitz LE, *et al.*** Declines in HPV vaccine type prevalence in women screened for cervical cancer in the United States: Evidence of direct and herd effects of vaccination. *Vaccine* 2019; **37**: 3918–3924.

19. **Feiring B, *et al.*** Substantial decline in prevalence of vaccine-type and nonvaccine-type human papillomavirus (HPV) in vaccinated and unvaccinated girls 5 years after implementing HPV vaccine in Norway. *The Journal of Infectious Diseases* 2018; **218**: 1900–1910.

20. **Guiqian Z, *et al.*** Human papillomavirus infection rates before and after the introduction of prophylactic vaccines in Kunming, Yunnan, China. *Indian Journal of Medical Microbiology* 2020; **38**: 66–71.

21. **Kahn JA, *et al.*** Vaccine-type human papillomavirus and evidence of herd protection after vaccine introduction. *Pediatrics* 2012; **130**: e249–e256.

22. **Kahn JA, *et al.*** Substantial decline in vaccine-type human papillomavirus (HPV) among vaccinated young women during the first 8 years after HPV Vaccine introduction in a community. *Clinical Infectious Diseases* Oxford Academic, 2016; **63**: 1281–1287.

23. **Kavanagh K, *et al.*** Introduction and sustained high coverage of the HPV bivalent vaccine leads to a reduction in prevalence of HPV 16/18 and closely related HPV types. *British Journal of Cancer* Nature Publishing Group, 2014; **110**: 2804–2811.

24. **Kavanagh K, *et al.*** Changes in the prevalence of human papillomavirus following a national bivalent human papillomavirus vaccination programme in Scotland: a 7-year cross-sectional study. *The Lancet. Infectious Diseases* 2017; **17**: 1293–1302.

25. **Cameron RL, *et al.*** Human papillomavirus prevalence and herd immunity after introduction of vaccination program, Scotland, 2009–2013. *Emerging Infectious Diseases* 2016; **22**: 56–64.

26. **Machalek DA, *et al.*** Very low prevalence of vaccine human papillomavirus types among 18- to 35-Year old australian women 9 years following implementation of vaccination. *The Journal of Infectious Diseases* 2018; **217**: 1590–1600.

27. **Markowitz LE, *et al.*** Prevalence of HPV after introduction of the vaccination program in the United States. *Pediatrics* 2016; **137**: e20151968.

28. **Oliver SE, *et al.*** Prevalence of human papillomavirus among females after vaccine introduction—National Health and Nutrition Examination Survey, United States, 2003–2014. *The Journal of Infectious Diseases* Oxford Academic, 2017; **216**: 594–603.

29. **Hirth JM, *et al.*** Regional variations in human papillomavirus prevalence across time in NHANES (2003-2014). *Vaccine* 2019; **37**: 4040–4046.

30. **McClung NM, *et al.*** Declines in Vaccine-Type Human Papillomavirus Prevalence in Females Across Racial/Ethnic Groups: Data From a National Survey. *The Journal of Adolescent Health: Official Publication of the Society for Adolescent Medicine* 2019; **65**: 715–722.

31. **Rosenblum HG, *et al.*** Declines in Prevalence of Human Papillomavirus Vaccine-Type Infection Among Females after Introduction of Vaccine - United States, 2003-2018. *MMWR. Morbidity and mortality weekly report* 2021; **70**: 415–420.

32. **McGregor S, *et al.*** Decline in prevalence of human papillomavirus infection following vaccination among Australian Indigenous women, a population at higher risk of cervical cancer: The VIP-I study. *Vaccine* 2018; **36**: 4311–4316.

33. **Mesher D, *et al.*** Reduction in HPV 16/18 prevalence in sexually active young women following the introduction of HPV immunisation in England. *Vaccine* 2013; **32**: 26–32.

34. **Mesher D, *et al.*** Continuing reductions in HPV 16/18 in a population with high coverage of bivalent HPV vaccination in England: an ongoing cross-sectional study. *BMJ open* 2016; **6**: e009915.

35. **Mesher D, *et al.*** The impact of the national HPV vaccination program in England using the bivalent HPV vaccine: surveillance of type-specific HPV in young females, 2010-2016. *The Journal of Infectious Diseases* 2018; **218**: 911–921.

36. **Purriños-Hermida MJ, *et al.*** Direct, indirect and total effectiveness of bivalent HPV vaccine in women in Galicia, Spain. *PloS One* 2018; **13**: e0201653.

37. **Söderlund-Strand A, Uhnoo I, Dillner J**. Change in population prevalences of human papillomavirus after initiation of vaccination: the high-throughput HPV monitoring study. *Cancer Epidemiology, Biomarkers & Prevention: A Publication of the American Association for Cancer Research, Cosponsored by the American Society of Preventive Oncology* 2014; **23**: 2757–2764.

38. **Sonnenberg P, *et al.*** Prevalence, risk factors, and uptake of interventions for sexually transmitted infections in Britain: findings from the National Surveys of Sexual Attitudes and Lifestyles (Natsal). *Lancet (London, England)* 2013; **382**: 1795–1806.

39. **Tabrizi SN, *et al.*** Fall in human papillomavirus prevalence following a national vaccination program. *The Journal of Infectious Diseases* 2012; **206**: 1645–1651.

40. **Tabrizi SN, *et al.*** Assessment of herd immunity and cross-protection after a human papillomavirus vaccination programme in Australia: a repeat cross-sectional study. *The Lancet Infectious Diseases* 2014; **14**: 958–966.

41. **Woestenberg PJ, *et al.*** Assessment of herd effects among women and heterosexual men after girls-only HPV16/18 vaccination in the Netherlands: A repeated cross-sectional study. *International Journal of Cancer* 2019; **144**: 2718–2727.

42. **Batmunkh T, *et al.*** HPV genoprevalence and HPV knowledge in young women in Mongolia, five years following a pilot 4vHPV vaccination campaign. *Papillomavirus Research (Amsterdam, Netherlands)* 2019; **8**: 100175.

43. **Berenson AB, Hirth JM, Chang M**. Change in human papillomavirus prevalence among U.S. women aged 18-59 years, 2009-2014. *Obstetrics and Gynecology* 2017; **130**: 693–701.

44. **Brogly SB, *et al.*** Human papillomavirus vaccination and cervical cytology in young minority women. *Sexually Transmitted Diseases* 2014; **41**: 511–514.

45. **Carozzi F, *et al.*** Monitoring vaccine and non-vaccine HPV type prevalence in the post-vaccination era in women living in the Basilicata region, Italy. *BMC infectious diseases* 2018; **18**: 38.

46. **Enerly E, *et al.*** An observational study comparing HPV prevalence and type distribution between HPV-vaccinated and -unvaccinated girls after introduction of school-based HPV vaccination in Norway. *PloS One* 2019; **14**: e0223612.

47. **Franceschi S, *et al.*** Urine testing to monitor the impact of HPV vaccination in Bhutan and Rwanda. *International Journal of Cancer* 2016; **139**: 518–526.

48. **Goggin P, *et al.*** Low prevalence of vaccine-type HPV infections in young women following the implementation of a school-based and catch-up vaccination in Quebec, Canada. *Human Vaccines & Immunotherapeutics* 2018; **14**: 118–123.

49. **Gray P, *et al.*** Occurrence of human papillomavirus (HPV) type replacement by sexual risk-taking behaviour group: Post-hoc analysis of a community randomized clinical trial up to 9 years after vaccination (IV). *International Journal of Cancer* 2019; **145**: 785–796.

50. **Gray P, *et al.*** Evaluation of HPV type-replacement in unvaccinated and vaccinated adolescent females—Post-hoc analysis of a community-randomized clinical trial (II). *International Journal of Cancer* 2018; **142**: 2491–2500.

51. **Grün N, *et al.*** Oral human papillomavirus (HPV) prevalence in youth and cervical HPV prevalence in women attending a youth clinic in Sweden, a follow up-study 2013-2014 after gradual introduction of public HPV vaccination. *Infectious Diseases (London, England)* 2015; **47**: 57–61.

52. **Guo F, Hirth JM, Berenson AB**. Comparison of HPV prevalence between HPV-vaccinated and non-vaccinated young adult women (20-26 years). *Human Vaccines & Immunotherapeutics* 2015; **11**: 2337–2344.

53. **Institut National de Santé Publique du Québec I**. *Prévalence des infections au virus du papillome humain (VPH) : résultats de l’étude PIXEL—Portrait de la santé sexuelle des jeunes adultes au Québec, 2013-2014*. Québec, 2015 Sep.

54. **Jeannot E, *et al.*** Prevalence of vaccine type infections in vaccinated and non-vaccinated young women: HPV-IMPACT, a self-sampling study. *International Journal of Environmental Research and Public Health* 2018; **15**: 1447.

55. **Kumakech E, *et al.*** Significantly reduced genoprevalence of vaccine-type HPV-16/18 infections among vaccinated compared to non-vaccinated young women 5.5 years after a bivalent HPV-16/18 vaccine (Cervarix®) pilot project in Uganda. *PloS One* 2016; **11**: e0160099.

56. **Lynge E, *et al.*** Prevalence of high-risk human papillomavirus after HPV-vaccination in Denmark. *International Journal of Cancer* 2020; **147**: 3446–3452.

57. **Murall CL, *et al.*** HPV cervical infections and serological status in vaccinated and unvaccinated women. *Vaccine* 2020; **38**: 8167–8174.

58. **Schlecht NF, *et al.*** Cervical, anal and oral HPV in an adolescent inner-city health clinic providing free vaccinations. *PloS One* 2012; **7**: e37419.

59. **Shilling H, *et al.*** Human papillomavirus prevalence and risk factors among Australian women 9-12 years after vaccine program introduction. *Vaccine* 2021; **39**: 4856–4863.

60. **Wendland EM, *et al.*** Effectiveness of a universal vaccination program with an HPV quadrivalent vaccine in young Brazilian women. *Vaccine* 2021; **39**: 1840–1845.

61. **Woestenberg PJ, *et al.*** Bivalent Vaccine Effectiveness Against Type-Specific HPV Positivity: Evidence for Cross-Protection Against Oncogenic Types Among Dutch STI Clinic Visitors. *The Journal of Infectious Diseases* 2018; **217**: 213–222.

62. **Wright TC, *et al.*** HPV infections and cytologic abnormalities in vaccinated women 21-34 years of age: Results from the baseline phase of the Onclarity trial. *Gynecologic Oncology* 2019; **153**: 259–265.
